# Effects of the ActiveHip+ mHealth intervention on the recovery of older adults with hip fracture and their family caregivers: a multicentre open-label randomised controlled trial

**DOI:** 10.1016/j.eclinm.2024.102677

**Published:** 2024-06-07

**Authors:** Rafael Prieto-Moreno, Marta Mora-Traverso, Fernando Estévez-López, Pablo Molina-Garcia, Mariana Ortiz-Piña, Susana Salazar-Graván, Víctor Cruz-Guisado, Marta Linares Gago, Miguel Martín-Matillas, Patrocinio Ariza-Vega

**Affiliations:** a Nursing Research, Innovation and Development Centre of Lisbon (CIDNUR), Nursing School of Lisbon (ESEL), Lisbon, Portugal; b SPORT Research Group (CTS-1024), CIBIS (Centro de Investigación para el Bienestar y la Inclusión Social) Research Center, University of Almería, Almería, Spain; c Biomedical Research Unit, Torrecárdenas University Hospital, 04009, Almería, Spain; d Department of Education, Faculty of Education Sciences, University of Almería, Almería, Spain; e PA-HELP “Physical Activity for HEaLth Promotion” Research Group, Department of Physical Education and Sports, Faculty of Sport Science, University of Granada, Granada, Spain; f Instituto de Investigación Biosanitaria ibs.Granada, Granada, Spain; g Department of Physiotherapy, Faculty of Health Science, University of Granada, Granada, Spain; h Department of Child and Adolescent Psychiatry/Psychology, Erasmus MC University Medical Centre, Rotterdam, the Netherlands; i Department of Social and Behavioral Sciences, Harvard T. H. Chan School of Public Health, Boston, MA, USA; j Department of Physical Therapy, Occupational Therapy, Rehabilitation and Physical Medicine, Faculty of Health Sciences, Rey Juan Carlos University, Madrid, Spain; k Orthopaedic Surgery and Traumatology Service, University Hospital Virgen de las Nieves, Granada, Spain; l Department of Physical Medicine and Rehabilitation, University Hospital Jerez de la Frontera, Cadiz, Spain; m Department of Physical Medicine and Rehabilitation, University Hospital Puerto Real, Cadiz, Spain; n PROFITH (PROmoting FITness and Health Through Physical Activity) Research Group, Department of Physical Education and Sports, Faculty of Sport Science, Sport and Health University Research Institute (iMUDS), University of Granada, Granada, Spain

**Keywords:** Digital health, Health literacy, Osteoporotic hip fracture, Patient education, Tele-rehabilitation

## Abstract

**Background:**

Mobile health (mHealth) systems are a promising alternative for rehabilitation of hip fracture, addressing constrained healthcare resources. Half of older adults fails to recover their pre-fracture routines, which imposes a burden on caregivers. We aimed to test the effectiveness of the 3-month ActiveHip + mHealth intervention on physical and psychological outcomes of older adults with hip fracture and their family caregivers.

**Methods:**

In a multicentre open-label randomised controlled trial conducted across 3 hospitals in Andalusia (Spain), patients older than 65 with a hip fracture, who were previously independent and lacked cognitive impairment were recruited alongside with their caregivers. Participants were randomly allocated (1:1) to the intervention group (ActiveHip+) or control (usual care) group. The intervention group underwent a 12-week health education and tele-rehabilitation programme through the ActiveHip + mHealth intervention. The primary outcome, physical performance, was assessed using the Short Physical Performance Battery at three time points: at hospital discharge (baseline), 3-month after surgery (post intervention) and 1-year after surgery follow-up. Primary analyses of primary outcomes and safety data followed an intention-to-treat approach. This study is registered at ClinicalTrials.gov, NCT04859309.

**Findings:**

Between June 1st, 2021 and June 30th, 2022 data from 105 patients and their caregivers were analysed. Patients engaged in the ActiveHip + mHealth intervention (mean 7.11 points, SE 0.33) showed higher physical performance compared with patients allocated in the control group (mean 5.71 points, SE 0.32) at 3 months after surgery (mean difference in change from baseline 1.40 points, SE 0.36; puncorrected = 0.00011). These benefits were not maintained at 1-year after surgery follow-up (mean difference in change from baseline 0.19 points, SE 0.47; puncorrected = 0.68). No adverse events, including falls and refractures, were reported during the tele-rehabilitation sessions. At 3-months, the intervention group had 2 falls, compared to 4 in the control group, with no observed refractures. At the 1-year follow-up, the intervention group experienced 7 falls and 1 refracture, while the control group had 13 falls and 2 refractures.

**Interpretation:**

This study suggests that the ActiveHip + mHealth intervention may be effective for recovering physical performance in older adults with hip fracture. Importantly, the implementation of ActiveHip + into daily clinical practice may be feasible and has already been adopted in 18 hospitals, mostly in Spain but also in Belgium and Portugal. Thus, ActiveHip + could offer a promising solution when rehabilitation resources are limited. However, its dependence on caregiver support and the exclusion of participants with cognitive impairment makes it necessary to be cautious about its applicability. In addition, the non-maintenance of the effectiveness at 1-year follow-up highlights the need of refinement the ActiveHip + intervention to promote long-lasting behavioural changes.

**Funding:**

10.13039/100014419EIT Health and the Ramón y Cajal 2021 Excellence Research Grant action from the 10.13039/501100004837Spanish Ministry of Science and Innovation.

**Research in context**

**Evidence before this study**

We searched PubMed with the keywords ((older adult) OR (family caregivers)) AND (hip fracture)) AND ((mHealth) OR (tele-rehabilitation)) OR (digital health)) OR (eHealth)) for articles published in English from database inception to February, 5th 2024. Out of the 93 search results, only 8 studies focused on digital interventions for older adults with hip fracture and their family caregivers. Existing literature indicates that tele-rehabilitation outperforms control groups in terms of physical performance and functional status. However, evidence on psychological outcomes remains scarce, particularly in aspects such as pain management, emotional status and even in some aspects such as fear of falling, where there is no previous evidence.

**Added value of this study**

To the best of our knowledge, this is the largest randomised controlled trial (RCT) in the field and includes a 1-year after surgery follow-up to measure whether long-lasting behavioural changes have occurred. This study suggests that the ActiveHip + mobile health (mHealth) intervention could potentially enhance physical performance recovery in older adults with hip fracture 3 months after surgery. The fact that this improvement was not maintained at the 1-year after surgery follow-up may indicate that a 3-month intervention period may not be sufficient to ensure sustainable behavioural changes. Additionally, it provides for the first time a comprehensive intervention for both, the older adult with hip fracture and their family caregivers. Importantly, this study emphasises the approach of co-creation in the development of mHealth interventions, involving key stakeholders such as older adults with hip fracture, family caregivers and health providers, underscoring its significance in bridging clinical practice with patient and caregiver needs. This study also contributes to provide solutions to the lack of resources in hip fracture rehabilitation, as the results suggest that the ActiveHip + mHealth intervention may lead to improvements in the recovery of older adults with hip fracture and their family caregivers.

**Implications of all the available evidence**

An mHealth intervention may improve the objective physical function and anxiety in older adults with hip fracture and the burden and depression in their family caregivers. Additionally, our work supports the use of digital health in the recovery of older adults with hip fracture by combining health education and tele-rehabilitation programmes. This approach offers a feasible and potentially effective rehabilitation option from home, minimising the need for extensive healthcare resources while tailoring care for hip fractures and enhancing accessibility. Limitations of the present study include the dependence of the ActiveHip + mHealth intervention on caregiver support and the exclusion of participants with cognitive impairments, which affects its scalability. Future studies could further optimise and evaluate the implementation of mHealth interventions in this population, including incorporating mHealth into daily clinical practice to meet patients' specific needs more accurately.

## Introduction

The incidence of hip fracture is high. In Europe, for instance, there are 250 fractures out of 100,000 people,^1^ a figure that is steadily increasing. Hip fracture negatively impacts physical and psychological outcomes in older adults.^2^ Hip fracture is the leading cause of morbidity in older adults,^3^ making older adults highly dependent involving a burden in their family caregivers. Collectively, there is a need to improve how the recovery of hip fracture is managed in older adults and their family caregivers.^4^

The main goal in the rehabilitation of a hip fracture is to recover the individual's pre-fracture routine,^4^ addressing both the physical and psychological impact of the fracture. After surgery, the muscles suffer a decrease in strength and mass, reducing older adults' physical performance and functional status,^5^ which is the ability to perform activities of daily living (ADLs). In addition, hip fracture imposes a significant psychological burden, with half of affected older adults experiencing anxiety^6^ and 23% depression.^7^ These factors can exacerbate pain,^6^ leading to delayed decreased mobility and lower participation in rehabilitation.^8^ As rehabilitation activities decreases, there is an associated increase in fear of falling, linked to lower social participation.^9^ Collectively, these factors contribute to a negative and generalised impact on the quality of life after a hip fracture.^10^ Therefore, it is essential to develop comprehensive health education and rehabilitation interventions addressing the impact of hip fracture.

Hip fracture also imposes a burden on older adults' family caregivers,^11^ who play a crucial role in providing physical and psychological support during the recovery.^12^ In fact, their support is vital immediately after hospital discharge, when older adults are highly dependent to be in their own home.^13^ Consequently, family caregivers often experience a burden characterised by increased levels of anxiety, depression and low back pain.^11^ Although caregiver's health status is directly associated with the potential for recovery of older adults with hip fracture,^14^ caregivers are often unrecognised by health providers.^12^ Thus, family caregivers seek more information to play a more active role in the decision-making process during their relatives' recovery.^15^

International guidelines for the management of hip fracture highlight the importance of multidisciplinary approaches^6^ and early intervention including rehabilitation and health eduaction.^16^ However, older adults with hip fracture are not usually offered such programmes after hospital discharge because of the constrained healthcare system resources.^17^ Thus, it is a clinical priority to improve accessibility to rehabilitation of hip fracture.^17^ Mobile health (mHealth), the use of mobile devices to support health promotion, appears promising due to its widespread accessibility compared to traditional in-person rehabilitation.^18^ mHealth has evolved from traditional procedures such as websites or recorded videos^2^^,^^19^ to mobile apps.^20^^,^^21^ To our knowledge, there are only 2 randomised controlled trials (RCTs)^20^^,^^21^ suggesting that mHealth systems may have positive effects in the recovery of the older adults with hip fracture.^22^ These studies were a 3-week pilot RCT study (*n* = 31)^20^ and a 3-month RCT study (*n* = 58).^21^ However, these interventions were conducted in a single centre and did not provided specific contents for family caregivers, despite their key role during the recovery. We developed ActiveHip+, an mHealth intervention of 3 months that comprises both an app to provide a tele-rehabilitation and health education programmes for older adults with hip fracture and their family caregivers and a webpage for health providers to follow-up the older adults with hip fracture. To overcome limitations of prior RCTs, we included 110 older adults and their corresponding family caregivers from three hospitals and we involved both older adults and family caregivers, considering the key role the latter play in this process. Therefore, the aim of this study was to experimentally test the effectiveness of the ActiveHip + mHealth intervention on physical and psychological outcomes in older adults with hip fracture and their family caregivers.

## Methods

### Study design

The present study is a multicentre RCT, conducted following the guidelines established by the Helsinki Declaration and Law 14/2007 on Biomedical Research. The older adults with hip fracture and their family caregivers involved in the study were recruited in three public hospitals in Andalucía (southern Spain): Virgen de las Nieves University Hospital (Granada), Jerez de la Frontera University Hospital (Cádiz) and Puerto Real University Hospital (Cádiz). The ActiveHip + project was approved by the Ethics Committee of the Research Centre of Granada (CEI-GRANADA, 21/07/2021) and it was also pre-registered in Clinicaltrials.gov (NCT04859309). In addition, the present study adheres to the CONSORT 2010 Statement as part of the EQUATOR reporting guidelines, ensuring transparency and clarity in trial reporting. The primary endpoint of the study was change in objectively measured physical performance measured through the Short Physical Performance Battery (SPPB) at 3-months after surgery. The secondary endpoint was the change in the rest of the outcomes described in the protocol.^23^

### Participants

Between June 1st, 2021 and June 30th, 2022, a total of 110 older adults with hip fracture and their family caregivers who met the inclusion criteria were recruited to participate in the present study during the hospital stay. Once participants receive an explanation from the research team about the main characteristics of the programme and signed the informed consent, they were randomly allocated to the intervention group (*n* = 55) or control group (*n* = 55). The inclusion criteria were: (i) to have a hip fracture; (ii) to be, at least, 65 years of age; (iii) to have a high prefracture functional status a week before surgery (i.e., Functional Independence Measure (FIM) overall score >90 points); (iv) to be allowed weight-bearing at 48 h after the surgery; (v) to be discharged to their own home or to a relative's home; (vi) to have a family caregiver with Internet access; and (vi) to read, understand and sign the informed consent. The exclusion criteria were: (i) to have severe cognitive impairment (Pfeiffer test score >4); (ii) to be discharged to a nursing home; or (iii) to have postoperative complications that prevent the start of rehabilitation in the first postoperative week.

### Randomisation and masking

Randomisation to the intervention or control group was done through sealed, numbered envelopes. Specifically, a designated health provider from each of the three study hospitals, who was not involved in participant recruitment, assessment or data analysis, managed the allocation process. These providers were contacted to open an envelope when an older adult with hip fracture and their family caregiver from their respective hospital agreed to participate.

In terms of participants masking, they were not blinded as they were aware of their participation in rehabilitation through the ActiveHip + mHealth intervention. To mitigate potential bias in the assessment process, assessors evaluating participants were blinded to participants allocation.

### Procedures

A part of the intervention was common to the older adults recruited to this study, irrespective of which group they belonged to, the tele-rehabilitation or the control group. During the hospital stay, all older adults underwent the usual care established by the Andalusian Public Healthcare System for a hip fracture, which is composed of 3–5 rehabilitation sessions during weekdays (no weekends). In addition, all participants were given a booklet during their stay in the hospital with recommendations and some exercises to do during the first days of the recovery.

#### Intervention group

A detailed description of ActiveHip+ is provided elsewhere.^23^ Briefly, older adults allocated in this group used ActiveHip+, an mHealth intervention composed of occupational therapy, physical exercise and health education for older adults with hip fracture supported by their family caregivers. This intervention is delivered through a mobile app linked to a webpage for health providers to follow-up. The app had a set of general recommendations and an on-demand daily life activities section to be used when needed. We aimed to mitigate the decline in the intrinsic capacity of older adults with hip fracture, which is highly associated with ageing. During 3 months, older adults with hip fracture could perform three video-recorded sessions each week (two of physical exercise and one of occupational therapy) supported by their family caregivers.^23^ Both the physical exercise and the occupational therapy programme were divided in four levels of difficulty, based on the older adults with hip fracture's physical and functional status evolution. The participants were contacted regularly so as to solve possible doubts about the use of the ActiveHip + intervention, a strategy established considering the lack of digital literacy of participants in this study. In addition, this follow-up allowed health providers to adapt the tele-rehabilitation programme to the progression of each participant, aiming at individualise the intervention. To this end, different progression criteria were established and it was included an option in the website to adapt the pace of the programme, evaluating the starting level of each person and their evolution after each tele-rehabilitation session.

Importantly, our intervention was designed following the Model of Human Occupations (MOHO)^24^ and World Health Organisation guidelines for healthy aging.^25^ ActiveHip + aimed to provide a training programme but also to increase patients’ health literacy to ensure that they are able to interact and respond successfully to the needs of their daily context and environment^24^ (i.e., functional capacity),^25^ despite of the existing intrinsic decline. ActiveHip+ was co-created with main stakeholders, including older adults with hip fracture, family caregivers and health providers.^26^ Including stakeholders allowed us to better contextualise and adapt our digital intervention to these patients such as their skills in using digital tools (i.e., digital literacy), which are critical barriers to the use of digital health. Thus, our approach was in line with guidelines for developing comprehensive and multidisciplinary interventions that aim to improve several outcomes or require skills on the part of those providing and receiving the intervention^27^ An educational programme for their family caregivers was also provided through the app. Five modules were available to older adults and seven modules were available to family caregivers with information on the recovery and prevention tips. To provide a holistic intervention, the educational programme offered information on different topics (e.g., medication, diet or tips to prevent falls and secondary fractures) beyond physical rehabilitation.

#### Control group

The participant assigned to the control group, received the usual rehabilitation offered so far by the Andalucian Public HealthCare System. It was composed of 5–15 face-to-face sessions delivered by physiotherapists or occupational therapist at patient's home. These sessions focused on general recommendations to improve balance and functional status. Furthermore, participants allocated to this group were provided with an informative booklet with recommendations on physical exercise to do at home.

### Outcomes

Detailed information is available in the Appendix 1. A brief description is provided as follow. Outcomes were evaluated at three time points: hospital discharge (baseline), 3-month after surgery (post intervention) and 1-year after surgery follow-up. The primary goal of our intervention was to aid in the recovery of patients during the sub-acute phase of their hip fracture (typically, 3-month after hospital discharge). Although not included in the protocol, a 1-year follow-up after surgery was also to evaluate whether the potential benefits of the intervention are maintained in the long-term after ActiveHip + intervention cessation.

**Sociodemographic variables** (e.g., gender, age, place of residence) were collected during the interview with older adults with hip fracture and family caregivers. We collected clinical data (e.g., type of fracture, type of surgery and hospital stay) from the participants’ clinical record.

**Older adults with hip fracture outcomes** The primary outcome of this study was objectively measured physical performance measured using the Short Physical Performance Battery (SPPB) and Handgrip dynamometry. Secondary outcomes were self-reported functional status using the Functional Independence Measure (FIM) and self-reported pain using a 0 (no pain) to 10 (worst possible pain) numeric rating scale (NRS).

**Common outcomes for older adults with hip fracture and family caregivers** Quality of life, fear of falling and emotional status were self-reported using the EuroQol Quality of Life questionnaire (EQ-5D), Short Falls Efficacy Scale-International (SFES-I) and Hospital Anxiety and Depression Scale (HADS), respectively.

**Caregivers outcomes** The burden on caregivers, low back pain, and self-reported fitness were self-reported using the Caregiver Strain Index, Oswestry Disability Index questionnaire and International Fitness Scale (IFIS), respectively.

Importantly, monitoring of adverse events was conducted using self-reported questionnaires and patients' medical records from baseline to the 1-year after-surgery follow-up assessment (i.e., endpoint of follow-up for this study) in both groups. Adverse events for this study included falls and refractures.

### Statistical analysis

#### Sample size

We used the G∗Power software (Franz Faul, Christian-Albrechts-Universitätzu Kiel) (version 3.0.1)^28^ to estimate the sample size required, which was based on an a priori calculation that considered changes in physical performances from our previous pilot study.^29^ A total of 104 participants (52 participants per group) were required to reach statistical power. This calculation incorporated an anticipated statistical power of 80%, a type I error rate (alpha) of 5%, and a projected participant attrition rate of 15%.

The normal distribution of the data was checked with the Kolmogorov–Smirnov test. Descriptive characteristics of the sample are presented as mean and standard deviation (SD) or frequency and percentage when appropriate. Baseline differences between groups were tested using an independent sample t-test for continuous variables and a χ2 test or Mann–Whitney U test for categorical binomial and polynomial, respectively. The main effects of the ActiveHip+ were tested with the intention-to-treat approach. The criteria of the per-protocol approach, shown in the supplementary material were: (1) to have valid data in both pre- and post-intervention assessments and (2) to have accessed to the app at least half of the 84 days that the health educational and tele-rehabilitation programme delivered through the ActiveHip + app lasts.

Despite it was described in the protocol that the analysis will be through analysis of covariance (ANCOVA), the effects of the ActiveHip + intervention were tested using constrained baseline longitudinal analysis via a linear mixed model using the ‘LMMstar’ R-package.^30^ The dependent variable were outcomes included at three-time points: baseline, 3-month, and 1-year after surgery follow-up. The independent variables were the intervention option (ActiveHip + mHealth vs Control Group), time (baseline, 3-month and 1-year after surgery follow-up), and rehabilitation-by-time interaction. Data were presented as means and differences in the mean changes with standard error (SE) as an indicator of variance. The adequacy of the models was investigated via the predicted values and residuals. We examined linearity, representing a linear dose–response relationship, by treating each rehabilitation category as a continuous variable in the main model and confirming it through visual inspections.

Per-protocol analyses are presented as supplementary material and followed the same procedure as the explained above for the intention-to-treat analyses. For the intention-to-treat approach, all participants (*n* = 105) were included. Missing data were handled through a listwise deletion approach. In sensitivity analyses, presented as supplementary material, missing data were imputed using multiple imputation generating 10 iterations and 5 datasets, which were then averaged to obtain the imputed values for the intention-to-treat analyses.

All analyses were performed using IBM SPSS Statistics (SPSS, IBM Corporation version 25.0; Armonk, NY) and the software R version 4.3.1 and RStudio version 2023.09.0 + 463. We controlled for multiple testing by calculating false discovery rate (FDR) value utilising the Benjamin–Hochberg method^31^ via the ‘P adjust’ function from the ‘stats’ R package. This control was applied by type of analysis, time point and participant group. For instance, for the intention-to-treat analysis in patients, we adjusted the FDR for 19 outcomes at the 3-month follow-up. In this study, P_uncorrected_ <0.05 or P_FDR_ <0.05 was considered statistically significant, and all statistical tests were two-tailed tests.

### Role of the funding source

The funder of the study had no role in study design, data collection, data analysis, data interpretation, or writing of the report. RP-M and PA-V have access to and verify the underlying study data and were responsible for the decision to submit for publication.

## Results

**Fig. 1:**
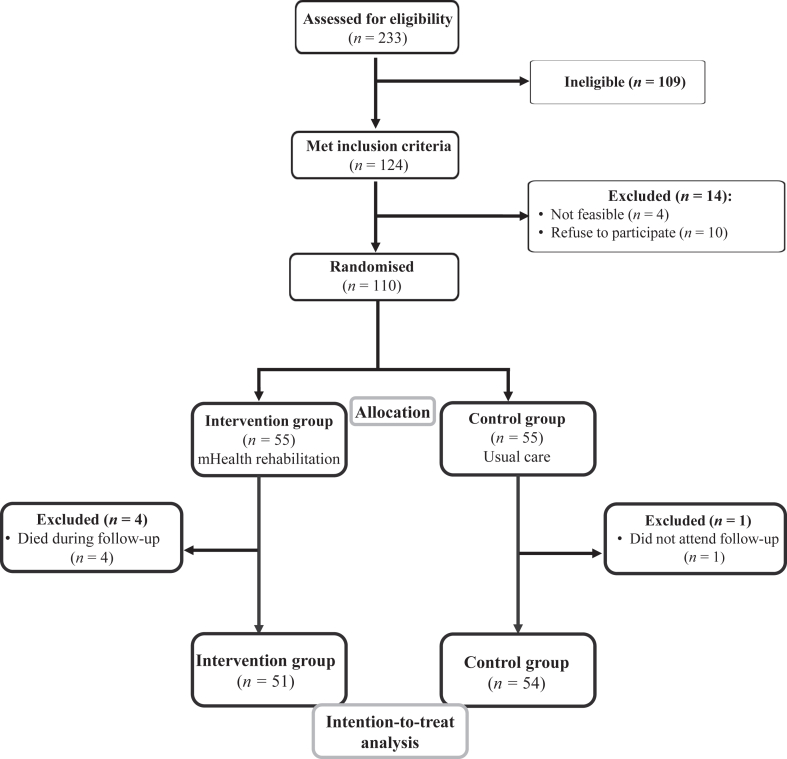
Consort flowchart of the sample recruited for this study. Description: This image details the recruitment and allocation process, including number of participants assessed for eligibility, those excluded and the reasons for their exclusion, the allocation of participants to different intervention groups, and the follow-up and analysis stages, including any losses to follow-up.

Between June 1st, 2021 and June 30th, 2022, 233 older adults with fracture were admitted to the hospitals included in this study to undergo hip surgery. Out of these 233, 124 meet the inclusion criteria and were invited to participate in this study, 110 accepted to use ActiveHip + intervention. Half of them were randomly allocated to intervention group and half of them to the control group. In the intervention group, out of these 55 participants who accepted, 51 older adults with hip fracture were included in the final analysis. In the control group, 54 were included. The exclusion and dropouts of older adults in both groups are detailed in the CONSORT 2021 flowchart (Fig. 1).

Table 1 shows the sociodemographic characteristics of the participants by allocation groups. There were no statistically significant differences in any of the outcomes included.

**Table 1:** Baseline characteristics of participants included in intention-to-treat analyses.

Variable	Intervention group (*n* = 51)	Control group (*n* = 54)
Age (years), mean (SD)	79.55 (7.11)	80.07 (7.74)
Sex, *n* (%)		
Women	37 (73)	38 (70)
Men	14 (27)	16 (30)
Type of injury, *n* (%)		
Fracture cervical femoral (Intracapsular)	29 (57)	30 (56)
Fracture trochanteric (Extracapsular)	22 (43)	24 (44)
Type of surgery, *n* (%)		
Prosthesis	16 (65)	13 (24)
Screw plate	33 (31)	36 (67)
PFN-A nail	2 (4)	5 (9)
Falls in the previous year, *n* (%)		
Yes	16 (31)	18 (33)
No	35 (69)	36 (67)
Pre-fracture residence, *n* (%)		
Own home	49 (96)	54 (100)
Nursing or relative's home	2 (4)	0 (0)
Post-fracture residence, *n* (%)		
Own home	45 (88)	47 (87)
Nursing or relative's home	6 (12)	7 (13)
Hospital stay (days), Mean (SD)	6.76 (4.1)	5.6 (3.3)
**Older adults' outcomes**		
Objectively measured physical performance (SPPB 0–12)	2.76 (0.97)	2.63 (1.22)
Functional status (FIM, 18–126)	79.05 (17.10)	80.47 (14.40)
Emotional status (HADS, 0–42)	15.59 (5.095)	15.04 (5.78)
Pain level (NRS, 0–10)	5.92 (2.2)	6.52 (1.78)
Fear of falling (SFES-I, 7–28)	19.54 (7.1)	20.02 (5.15)
Quality of life (EQ5D −0.65 to 1)	0.20 (0.31)	0.35 (0.49)
**Family caregivers' outcomes**		
Caregivers burden (CSI, 0–13)	5.98 (1.88)	6.72 (1.83)
Emotional status (HADS, 0–42)	11.49 (3.86)	12.90 (3.59)
Low back pain (ODI, 0–50)	10.92 (13.51)	9.00 (12.56)
Quality of life (EQ5D, −0.65 to 1)	0.80 (0.34)	0.85 (0.21)
Fear of falling (SFES-I, 7–28)	21.04 (6.79)	20.67 (5.72)
Self-reported fitness (IFIS, 4–20)	17.27 (3.49)	17.02 (3.24)

Values are Mean (SD) unless otherwise indicated. CSI, Caregivers' Strain Index; EQ5D, EuroQol-5D; FIM, Functional Independence Measure; HADS, Hospital Anxiety and Depression Scale; IFIS, International Fitness Scale; NRS, Numeric rating scale; ODI, Oswestry Low Back Disability; PFN-A, Proximal Femoral Nail; SD, standard deviation; SFES-I, Short Falls Efficacy Scale; SPPB, Short Physical Performance Battery.

The normality of residuals prior to conducting the linear mixed model showed that the residuals followed a normal distribution across all variables, unless EQ5D total index for caregivers, where the over-dispersed data indicates that a substantial majority of caregivers reported a maximal quality of life score. This is shown in Appendix 2 (Figures S1 and S2). The main analyses are presented in Table 2 for older adults with fracture and in Table 3 for family caregivers. Both tables show the constrained linear mixed model between the intervention group and control group at baseline (reference), 3-month and 1-year after surgery follow-up.

**Table 2:** Differences in older adults’ outcomes between groups at 3-month after surgery (post intervention) and at 1-year follow-up: intention-to-treat analyses.

Measurement	Month	Intervention group (ActiveHip + mHealth)			Control group (Usual care)			Differences in change from baseline (ActiveHip + minus Usual care), Mean (SE)	*p*_uncorrected_	*p*_FDR_
		*n*	Mean (SE)	Change from baseline, mean (SE)	*n*	Mean (SE)	Change from baseline, mean (SE)			
Objectively measured physical performance (SPPB, 0–12)	0	51	2.69 (0.11)	Reference	54	2.69 (0.11)	Reference	Reference	Reference	Reference
	3	51	7.11 (0.33)	4.43 (0.34)	54	5.71 (0.32)	3.02 (0.33)	1.40 (0.36)	**0.00011**	**0.0021**
	12	31	6.34 (0.35)	3.66 (0.35)	29	5.18 (0.34)	3.49 (0.35)	0.19 (0.47)	0.68	0.99
Balance (SPPB, 0–4)	0	51	1.48 (0.06)	Reference	54	1.48 (0.06)	Reference	Reference	Reference	Reference
	3	51	3.59 (0.10)	2.11 (0.12)	54	3.18 (0.09)	1.70 (0.12)	0.40 (0.16)	**0.013**	**0.041**
	12	31	4.03 (0.18)	2.56 (0.20)	29	3.91 (0.18)	2.44 (0.19)	0.08 (0.19)	0.67	0.99
Gait speed (SPPB, 0–4)	0	51	0.68 (0.05)	Reference	54	0.68 (0.05)	Reference	Reference	Reference	Reference
	3	51	1.76 (0.13)	1.08 (0.14)	54	1.26 (0.13)	0.58 (0.14)	0.49 (0.13)	**0.00042**	**0.0040**
	12	31	1.91 (0.08)	0.51 (0.09)	29	1.27 (0.05)	0.59 (0.09)	−0.05 (0.17)	0.76	0.99
Chair stand (SPPB, 0–4)	0	51	1.42 (0.04)	Reference	54	1.42 (0.04)	Reference	Reference	Reference	Reference
	3	51	1.77 (0.14)	1.36 (0.14)	54	1.26 (0.13)	0.85 (0.14)	0.51 (0.17)	**0.0023**	**0.0109**
	12	31	1.86 (0.19)	1.45 (0.20)	29	1.76 (0.20)	1.34 (0.21)	0.21 (0.22)	0.36	0.99
Handgrip strength kg	0	51	18.20 (0.51)	Reference	54	18.20 (0.51)	Reference	Reference	Reference	Reference
	3	49	20.40 (0.50)	2.26 (0.49)	52	19.90 (0.46)	1.72 (0.47)	0.57 (0.65)	0.38	0.52
	12	28	20.14 (0.95)	1.94 (0.58)	28	19.16 (0.59)	1.41 (0.59)	0.63 (0.84)	0.45	0.99
Functional status (FIM, 18–126)	0	51	77.30 (1.51)	Reference	54	77.30 (1.51)	Reference	Reference	Reference	Reference
	3	51	114.50 (1.34)	37.20 (1.76)	54	110.80 (1.30)	33.50 (1.73)	3.36 (2.65)	0.21	0.38
	12	36	107.90 (2.73)	30.54 (3.00)	37	105.80 (2.67)	28.47 (2.94)	2.60 (3.15)	0.41	0.99
FIM self-care (6–42)	0	51	19.00 (0.54)	Reference	54	19.00 (0.54)	Reference	Reference	Reference	Reference
	3	51	36.80 (0.70)	17.74 (0.81)	54	34.60 (0.68)	15.52 (0.80)	2.16 (1.08)	**0.047**	0.103
	12	36	34.90 (1.11)	15.85 (1.19)	37	33.60 (1.10)	14.52 (1.17)	1.26 (1.29)	0.33	0.99
FIM sphincter (2–14)	0	51	11.30 (0.34)	Reference	54	11.30 (0.34)	Reference	Reference	Reference	Reference
	3	51	13.30 (0.14)	2.08 (0.27)	54	12.90 (0.14)	1.64 (0.27)	0.44 (0.36)	0.22	0.38
	12	36	12.90 (0.29)	1.61 (0.38)	37	12.10 (0.29)	0.87 (0.38)	0.71 (0.43)	0.10	0.95
FIM transfer (3–21)	0	51	8.40 (0.56)	Reference	54	8.40 (0.56)	Reference	Reference	Reference	Reference
	3	51	18.40 (0.31)	9.96 (0.56)	54	17.80 (0.31)	9.40 (0.57)	0.34 (0.81)	0.67	0.75
	12	36	17.00 (0.62)	8.61 (0.82)	37	17.20 (0.61)	8.79 (0.81)	−0.09 (0.97)	0.92	0.99
FIM locomotion (2–14)	0	51	5.19 (0.34)	Reference	54	5.19 (0.34)	Reference	Reference	Reference	Reference
	3	51	11.99 (0.23)	6.80 (0.37)	54	11.51 (0.22)	6.32 (0.37)	0.43 (0.52)	0.42	0.53
	12	36	11.03 (0.45)	5.84 (0.55)	37	11.53 (0.44)	6.34 (0.54)	−0.27 (0.63)	0.67	0.99
FIM communication (2–14)	0	51	13.50 (0.09)	Reference	54	13.50 (0.09)	Reference	Reference	Reference	Reference
	3	51	13.90 (0.08)	0.34 (0.12)	54	13.70 (0.08)	0.16 (0.12)	0.13 (0.20)	0.50	0.59
	12	36	13.30 (0.24)	−0.26 (0.25)	37	12.90 (0.23)	−0.68 (0.24)	0.44 (0.23)	0.062	0.95
FIM psychosocial (3–21)	0	51	19.90 (0.19)	Reference	54	19.90 (0.19)	Reference	Reference	Reference	Reference
	3	51	20.30 (0.21)	0.39 (0.27)	54	20.20 (0.20)	0.33 (0.26)	0.02 (0.38)	0.97	0.97
	12	36	19.40 (0.42)	−0.54 (0.44)	37	18.80 (0.41)	−1.06 (0.43)	0.65 (0.45)	0.15	0.95
Emotional status (HADS, 0–42)	0	51	15.30 (0.57)	Reference	54	15.30 (0.57)	Reference	Reference	Reference	Reference
	3	51	11.60 (0.65)	−3.65 (0.76)	54	14.10 (0.63)	−1.20 (0.75)	−2.38 (1.00)	**0.018**	**0.049**
	12	31	15.30 (0.78)	−0.03 (0.99)	37	15.40 (0.77)	0.13 (0.97)	0.08 (1.21)	0.95	0.99
HADS anxiety (0–21)	0	51	7.66 (0.49)	Reference	54	7.66 (0.41)	Reference	Reference	Reference	Reference
	3	51	4.03 (0.49)	−3.62 (0.56)	54	6.51 (0.47)	−1.15 (0.55)	−2.48 (0.73)	**0.0010**	**0.0063**
	12	35	5.66 (0.56)	−1.99 (0.71)	37	5.90 (0.55)	−1.76 (0.69)	−0.02 (0.87)	0.99	0.99
HADS depression (0–21)	0	51	7.61 (0.25)	Reference	54	7.61 (0.24)	Reference	Reference	Reference	Reference
	3	51	7.66 (0.35)	−0.01 (0.36)	54	7.54 (0.31)	−0.07 (0.35)	0.07 (0.46)	0.87	0.92
	12	35	7.61 (0.39)	1.95 (0.45)	37	9.52 (0.38)	1.91 (0.45)	0.03 (0.55)	0.96	0.99
Pain (NRS, 0–10)	0	51	6.23 (0.20)	Reference	54	6.23 (0.20)	Reference	Reference	Reference	Reference
	3	51	1.29 (0.28)	−4.94 (0.34)	54	2.16 (0.27)	−4.07 (0.34)	−0.79 (0.40)	**0.049**	0.103
	12	36	2.02 (0.37)	−4.21 (0.42)	37	2.11 (2.11)	−4.11 (0.41)	−0.13 (0.48)	0.79	0.99
Fear of falling (SFES, 7–28)	0	51	19.80 (0.60)	Reference	54	19.80 (0.60)	Reference	Reference	Reference	Reference
	3	51	12.10 (0.78)	−7.73 (0.95)	54	13.30 (0.76)	−6.51 (0.94)	−1.18 (1.11)	0.29	0.44
	12	36	13.30 (0.89)	−6.52 (1.02)	37	13.10 (0.87)	−6.68 (1.00)	0.10 (1.32)	0.94	0.99
Quality of life (EQ5D, −0.65 to 1)	0	51	0.30 (0.05)	Reference	54	0.30 (0.05)	Reference	Reference	Reference	Reference
	3	51	0.49 (0.06)	0.19 (0.07)	54	0.32 (0.05)	0.02 (0.07)	0.09 (0.09)	0.30	0.44
	12	35	0.49 (0.07)	0.19 (0.08)	37	0.54 (0.07)	0.24 (0.08)	−0.05 (0.10)	0.64	0.99
Self-perceived health (EQ5D-VAS, 0–100)	0	51	54.64 (2.21)	Reference	54	56.64 (2.21)	Reference	Reference	Reference	Reference
	3	51	80.00 (2.18)	25.42 (2.69)	54	72.22 (2.06)	17.53 (2.64)	9.15 (3.51)	**0.0096**	**0.036**
	12	35	73.23 (2.22)	17.72 (3.30)	37	69.29 (2.84)	15.24 (3.26)	3.29 (4.17)	0.42	0.99

EQ5D, EuroQol-5D; FDR, False Discovery Rate; FIM, Functional Independence Measure; HADS, Hospital Anxiety and Depression Scale; HG, Handgrip; *n*, sample size; NRS, Numeric rating scale; SE, Standard Error SFES-I, Short Falls Efficacy Scale; SPPB, Short Physical Performance Battery. Significant differences (p < 0.05) are highlighted in bold.

**Table 3:** Differences in family caregivers' outcomes between groups at 3-month after surgery (post intervention) and at 1-year follow-up: intention-to-treat analyses.

Outcome	Months	Intervention group (ActiveHip + mHealth)			Control group (Usual care)			Differences in change from baseline (ActiveHip + vs. usual care), mean (SE)	*p*_uncorrected_	*p*_FDR_
		*n*	Mean (SE)	Change from baseline, mean (SE)	*n*	Mean (SE)	Change from baseline, mean (SE)			
Caregiver’ burden (CSI, 0–13)	0	51	6.36 (0.18)	Reference	54	6.36 (0.18)	Reference	Reference	Reference	Reference
	3	51	3.64 (0.34)	−2.72 (0.35)	54	4.59 (0.33)	−1.77 (0.34)	0.95 (0.46)	**0.038**	0.17
	12	34	3.79 (0.52)	−2.57 (0.54)	31	3.89 (0.54)	−2.47 (0.56)	0.01 (0.57)	0.99	0.99
Emotional status (HADS, 0–42)	0	51	12.44 (0.42)	Reference	54	12.44 (0.42)	Reference	Reference	Reference	Reference
	3	51	9.24 (0.54)	−3.20 (0.68)	54	10.63 (0.52)	−1.18 (0.67)	1.29 (0.77)	0.096	0.29
	12	34	16.16 (0.62)	3.72 (0.74)	31	15.34 (0.65)	2.90 (0.76)	−0.77 (0.98)	0.43	0.83
HADS anxiety (0–21)	0	51	3.94 (0.31)	Reference	54	3.94 (0.31)	Reference	Reference	Reference	Reference
	3	51	2.07 (0.33)	−1.88 (0.46)	54	2.44 (0.32)	−1.51 (0.45)	0.27 (0.52)	0.60	0.77
	12	34	7.19 (0.40)	3.24 (0.50)	31	6.97 (0.42)	3.03 (0.51)	−0.16 (0.67)	0.81	0.91
HADS depression (0–21)	0	51	8.50 (0.20)	Reference	54	8.50 (0.20)	Reference	Reference	Reference	Reference
	3	51	7.18 (0.34)	−1.31 (0.38)	54	8.18 (0.33)	−0.32 (0.38)	1.00 (0.41)	**0.016**	0.14
	12	34	8.95 (0.31)	0.45 (0.36)	31	8.30 (0.32)	−0.20 (0.37)	−0.62 (0.53)	0.24	0.83
Quality of life (EQ5D, −0.65 to 1)	0	51	0.83 (0.03)	Reference	54	0.83 (0.03)	Reference	Reference	Reference	Reference
	3	51	0.90 (0.03)	0.07 (0.04)	54	0.86 (0.03)	0.04 (0.04)	−0.03 (0.05)	0.47	0.77
	12	34	0.85 (0.04)	0.02 (0.05)	31	0.87 (0.04)	0.05 (0.05)	0.03 (0.06)	0.60	0.90
Health today (EQ5D-VAS, 0–100)	0	51	73.20 (2.24)	Reference	54	73.20 (2.24)	Reference	Reference	Reference	Reference
	3	51	85.40 (1.46)	12.20 (2.45)	54	84.20 (1.42)	11.05 (2.43)	−1.27 (3.17)	0.70	0.79
	12	34	84.50 (1.69)	11.36 (2.79)	31	82.60 (1.73)	9.46 (2.82)	−2.96 (4.02)	0.46	0.83
Low back pain (ODI, 0–50)	0	51	9.94 (1.29)	Reference	54	9.94 (1.29)	Reference	Reference	Reference	Reference
	3	51	5.47 (1.43)	−4.48 (1.91)	54	6.61 (1.39)	−3.33 (1.88)	1.31 (2.35)	0.58	0.77
	12	34	10.67 (2.30)	0.72 (2.59)	31	13.49 (2.37)	3.54 (2.65)	3.55 (2.98)	0.23	0.83
Fear of falling (SFES-I, 7–28)	0	51	20.80 (0.60)	Reference	54	20.80 (0.60)	Reference	Reference	Reference	Reference
	3	51	11.20 (0.73)	−9.63 (0.91)	54	11.90 (0.71)	−8.92 (0.89)	0.76 (1.07)	0.48	0.77
	12	34	12.20 (0.89)	−8.63 (0.99)	31	11.40 (0.90)	−9.48 (1.00)	−0.45 (1.35)	0.74	0.91
Self-reported fitness (IFIS, 4–20)	0	51	17.10 (0.33)	Reference	54	17.1 (0.327)	Reference	Reference	Reference	Reference
	3	51	17.60 (0.40)	0.47 (0.48)	54	17.5 (0.394)	0.39 (0.47)	−0.08 (0.59)	0.89	0.89
	12	34	17.20 (0.47)	0.09 (0.56)	31	16.4 (0.490)	−0.73 (0.58)	−0.72 (0.74)	0.33	0.83

CSI, Caregivers' Strain Index; EQ5D, EuroQol-5D; FDR, False Discovery Rate; HADS, Hospital Anxiety and Depression Scale; IFIS, International Fitness Scale; *n*, sample size; ODI, Oswestry Low Back Disability; SE, Standard Error; SFES-I, Short Falls Efficacy Scale. Significant differences (p < 0.05) are highlighted in bold.

**Fig. 2:**
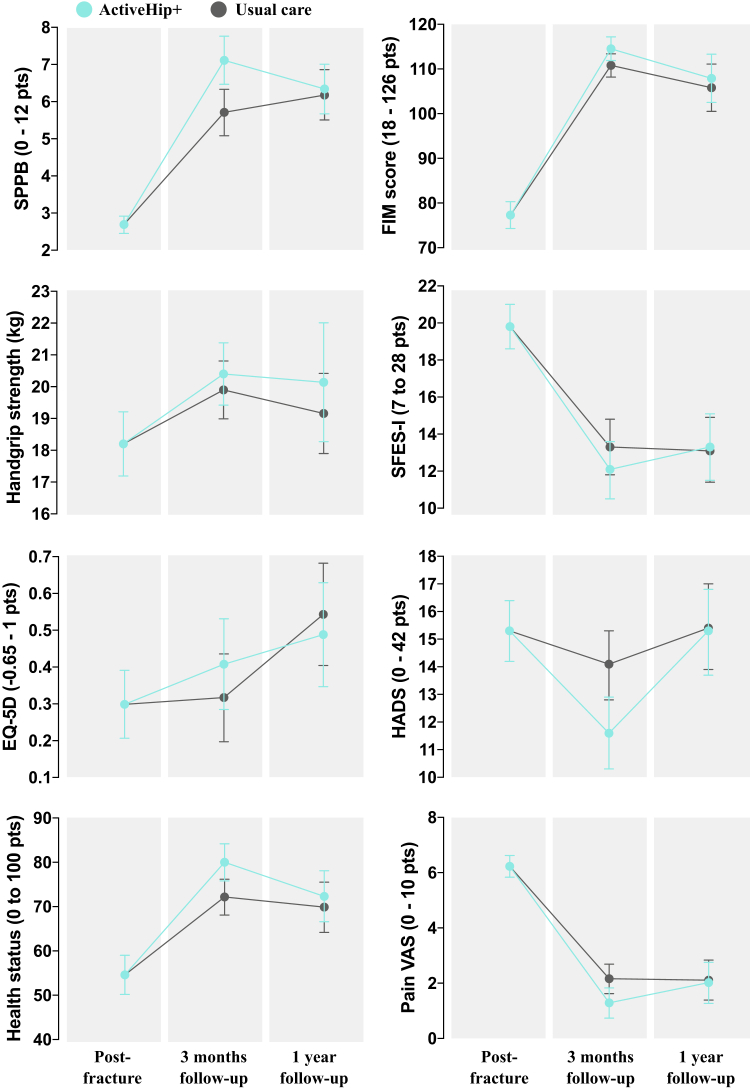
Changes in older adults' outcomes by time and group: intention-to-treat analyses. Description: Data points represent the model-estimated means and 95% confidence intervals (indicated by the I bars) from a constrained linear mixed model (cLMM) with baseline means constrained to be equal across study arms, reflecting the pre-randomisation nature of the baseline assessment.

At the 3-month post-fracture surgery follow-up (post intervention), older adults in the intervention group had a greater recovery in objectively measured physical performance (1.40 ± 0.36 points; puncorrected = 0.00011, pFDR = 0.0021), emotional status (−2.38 ± 1 points; puncorrected = 0.018, pFDR = 0.049), pain relief (−0.79 ± 0.34 points; puncorrected = 0.049, pFDR = 0.103) and self-perceived health (9.15 ± 3.51 points; puncorrected = 0.0096, pFDR = 0.036) than those in the control group. No effects were observed for the remaining older adults’ outcomes at 3-month follow-up (all, p > 0.20) and none of the previous effects were maintained at 1-year after surgery follow-up (all, p > 0.40). All these results are depicted in Table 2 and illustrated in Fig. 2.

**Fig. 3:**
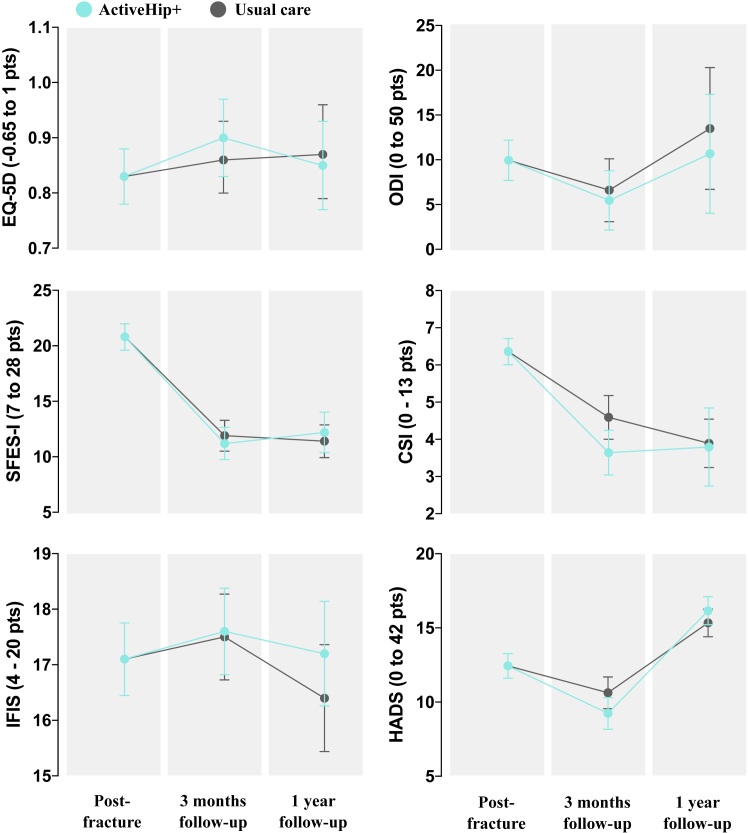
Changes in family caregivers' outcomes by time and group: intention-to-treat analyses. Description: Data points represent the model-estimated means and 95% confidence intervals (indicated by the I bars) from a constrained linear mixed model (cLMM) with baseline means constrained to be equal across study arms, reflecting the pre-randomisation nature of the baseline assessment.

Regarding family caregivers at the 3-month post-fracture surgery follow-up (post intervention), those in the intervention group had a greater decrease of the burden (−0.96 ± 0.46 points; puncorrected = 0.038, pFDR = 0.17) and depression status (−1.00 ± 0.41 points; puncorrected = 0.016, pFDR = 0.14) compared to controls. No effects were observed for the remaining caregivers’ outcomes at 3-month follow-up (all, p > 0.089) and none of the previous effects were maintained at 1-year after surgery follow-up (all, p > 0.23). All these results are depicted in Table 3 and illustrated in Fig. 3.

In general, findings from sensitivity analyses using multiple imputations for missing data were similar to intention-to-treat analyses. As exceptions, we only found differences in the pain relief at 3-months follow-up. Interestingly, the between-group difference observed in the intention-to-treat analyses, was not maintained at 3 months with the multiple imputations approach (−0.76 ± 0.40 points, p = 0.060) at 3 months. This is shown in Appendix 3 (Tables S4 and S5).

There were no adverse events related to the intervention. At three months after the surgery, there were 2 falls in the intervention group and 4 in the control group. Importantly, none of the falls in the intervention group occurred during the tele-rehabilitation sessions, and no refracture was observed. One year after the surgery, the intervention group had 7 falls and 1 refracture, while the control group had 13 falls and 2 refractures.

The per-protocol which represents the secondary analysis are shown in Appendix 3 (Figure S3, Tables S1–S3, Figures S4 and S5). Overall, effects size was similar to the results of the intention-to-treat approach.

## Discussion

In this RCT, we found that ActiveHip + may be effective to recover patients' objectively measured physical performance and to reduce anxiety and pain, but may have no effects on functional status, depression, fear of falling and quality of life. The ActiveHip + mHealth intervention also reduced family caregivers' burden and depression, but not fear of falling, low back pain, quality of life and self-reported fitness. None of these effects persisted at the 1-year after surgery follow-up, suggesting that a 3-month intervention may have been insufficient to promote long-lasting behavioural changes.

We found significant improvements in objective measures of physical function (i.e., performance-based test) among participants in the ActiveHip + intervention group compared to the control group. However, no effects were observed for subjective assessments of physical function (i.e., functional status by means of patients reported outcomes measures, PROMs). We speculate that this may be explained due to the lack of contents addressing some dimensions included in the FIM (i.e., sphincter, communication and psychosocial). This discordance in the findings for objectively and subjectively evaluated physical function might be related to the difference in the number of weekly sessions. Participants engaged in physical exercise sessions twice a week, primarily aimed at enhancing physical performance. Additionally, once a week, they participated in occupational therapy sessions focused on improving functional status through practice and repetition of ADLs, which is key in fostering people's belief about their capacity^32^ commonly known as self-efficacy. Thus, we speculate that a weekly session of occupational therapy may be insufficient for improving functional status of older adults with hip fractures, lacking enhancement of self-efficacy. Consequently, future interventions should include psychological components to improve the functional status, as a subjective assessment of physical function, while maintaining attention to the objective dimension of physical function.

In general, the ActiveHip + intervention had limited effects on psychological outcomes, with exceptions. Participants in the intervention group self-reported lower levels of anxiety than participants in the control group. The health education programme of the ActiveHip + focuses on increasing the older adults' ability to understand and use information to make health-related decisions (i.e., health literacy) and illustrates a roadmap of the typical journey for hip fracture recovery providing guidance to manage the new situation. This information and guidance may increase patients' awareness of their recovery resulting in lower anxiety,^33^ associated with uncertainties arising during hospitalisation (e.g., physical survival, life's disruption).^34^ The educational programme also encompasses information on pain self-management, which may have led to lowering the pain experienced by older adults with hip fracture in the experimental group.^35^ On the other hand, the ActiveHip + intervention may not have had effects on fear of falling, quality of life and depression in older adults with hip fracture. These outcomes may not have improved because they were not specifically targeted by the intervention. For instance, ActiveHip + may not have adequately addressed the improvement of self-efficacy or the pre-existing social isolation of older adults with hip fracture, which are closely related to these outcomes depression and diminished quality of life.^36^, ^37^, ^38^ In summary, future interventions may benefit from a more comprehensive psychosocial approach that specifically targets a broader range of outcomes in older adults with hip fracture, including improving the fostering of social relationships.

The present study demonstrates that the ActiveHip + intervention significantly reduced caregiver burden and depression 3 months after the intervention. We speculate that the addition of health education providing relevant information for caregivers during the recovery of older adults with hip fracture (e.g., strategies for promoting physical and mental well-being of caregivers or tips to prevent a secondary fracture), together with a section providing recommendations for ADLs, could have contributed to the reduction of caregiver burden. Offering education may empower caregivers to enhance their self-confidence in providing care, ultimately impacting some of these burden and depression-related aspects that were present just after the hip fracture occurred. The ActiveHip + intervention did not show significant improvements in low back disability, quality of life, and self-reported fitness level. When analysing the baseline scores of these outcomes, family caregivers in both groups reported low back pain, and their quality of life and self-reported fitness were optimal. Thus, the intervention may have reached a ceiling effect with these variables, and future interventions should consider other measures for caregivers such as objectively measured physical performance. Consistent with the patients’ results, the ActiveHip + intervention did not reduce caregivers' perceived fear of falling which was not specifically targeted by the intervention Therefore, further attention should be paid to this specific outcome in mHealth interventions, considering a holistic approach. Future digital health interventions for family caregivers should prioritise multicomponent approaches, including educational and psychological support.

Our findings may have clinical implications, as the ActiveHip + intervention offers a promising solution to the challenge of limited rehabilitation resources within healthcare systems. By facilitating early and sustainable recovery for patients, particularly in terms of enhancing physical performance and lowering anxiety and pain as well as supporting family caregivers by mitigating their burden and depression. Importantly, the implementation of ActiveHip + into daily clinical practice is remarkably feasible,^39^ it is being used in 14 hospitals in Spain, 3 in Belgium and one in Portugal. The digital and multidisciplinary nature of the ActiveHip+, combining physical exercise, occupational therapy and health education for patients along with specific modules for family caregivers, distinguishes it as a pioneering intervention. Despite ActiveHip+ is multidisciplinary, the constraints of limited resources and the imperative need of implementation in real clinical settings imposed limitations on its scope. Consequently, key aspects of recovery, such as psychological and nutritional interventions, unfortunately could only be addressed through the educational programme, potentially affecting our results in psychological outcomes and further research in more holistic interventions addressing these aspects is needed. Furthermore, in an ideal scenario without limited resources, future complex interventions such as digital health in the recovery of hip fracture should aim to be more dynamic involving monitoring the need of potential adaptations over time. This would involve an analysis of the interactions between the intervention provided, the users and their context. This approach may increase the possibility to respond more effectively to the patient's needs, which is essential in non-acute processes such as recovery from a hip fracture.

This study has limitations. First, the ActiveHip + mHealth intervention for the recovery of older adults with hip fracture may not be generalisable because participants need to (i) have the support of a family caregivers in overcoming patients' lack of skills in using mobile devices and (ii) not having cognitive impairment because it would make unfeasible to engage in the intervention. Second, we did not measure the participation of these family caregivers in the recovery, which may have influenced the older adults with hip fracture participation in rehabilitation. Third, the EQ5D total index for caregivers exhibited an over-dispersed distribution towards a maximal quality of life score among most of them. Consequently, the results should be interpreted with caution due to the possibility of a ceiling effect. Future studies should consider incorporating a quality-of-life assessment more tailored to caregivers, enabling the detection of significant changes attributable to the intervention. Fourth, the statistical analysis of the study was designed based on the primary outcome, which makes it necessary to take the results of the secondary variables cautiously due to the possible lack of statistical power. The main strengths of this study are its RCT design, relatively large sample size, inclusion of family caregivers and 1-year after surgery follow-up to test the maintenance of the intervention effects.

In conclusion, the ActiveHip + intervention may improve objectively measured physical performance and reduce anxiety and pain among older adults with hip fractures. However, it may have no effects on functional status, depression, fear of falling or quality of life. While the intervention may benefit family caregivers by reducing burden and depression, it may have no effects on low back pain, quality of life, fear of falling or self-reported fitness levels. The lack of sustained effects at the 1-year after surgery follow-up suggests that the 3-month intervention period may not be sufficient for promoting long-lasting behavioural changes.

## Contributors

PA-V led the development of this study. RP-M and MM-T recruited the sample. RP-M, MM-T and PM-G were responsible of data collection, monitoring and curation. RP-M and PM-G were responsible for data analysis. FE-L and PA-V secured funding for this study. RP-M, MM-T, PM-G, MO-P, SS-G, VC-G, MLG and PA-V collaborated in the development of the research protocol. RP-M, MM-T, FE-L, PM-G, MO-P, MM-M and PA-V designed the methodology of this study. RP-M and PA-V were responsible for project management and trial coordination. SS-G, VC-G, MLG and PA-V provided resources support. RP-M and MM-T wrote the original draft of this manuscript, supervised by FE-L and PA-V. PM-G, MO-P, SS-G, VC-G, MLG and MM-M reviewed and edited the manuscript. RP-M, MM-T, FE-L, PM-G, MM-M and PA-V have access to and verify the underlying study data. All authors had full access to all the data and final responsibility for the decision to submit for publication.

## Data sharing statement

Study protocol, statistical analysis plan, informed consent form and analytic code will be available immediately following publication to researchers who provide a methodologically sound proposal. Proposals must be sent to pariza@ugr.es to gain access. Data requestors will need to sign a data access agreement.

## Declaration of interests

All authors declare no competing interests.

## Appendix A Supplementary data

Supplementary data

TranslatedAbstract
