# Supplementary material for: Effects of the ActiveHip+ mHealth intervention on the recovery of older adults with hip fracture and their family caregivers: a multicentre open-label randomised controlled trial
Source: eClinicalMedicine. 2024 Jun 7;73:102677. doi: 10.1016/j.eclinm.2024.102677 (PMC11192790; doi:10.1016/j.eclinm.2024.102677)
Supplement: Supplementary data [file mmc1.docx]

# Appendix 1: Supplementary methods: Clinical outcomes for older adults with hip fracture and for family caregivers

Physical performance: It was assessed through the Short Physical Performance Battery (SPPB) and Handgrip dynamometry (HG). SPPB is a tool to evaluate lower extremity physical performance in older adults, including (i) balance (ii) gait speed and (iii) chair stand.^1^ The score ranged from 0 to 12 points, meaning a higher score better physical performance. The internal consistency is high, with Cronbach’s α= =0.87.^1^ HG is an indicator of physical function in older adults and an objective indicator of muscle strength.^2^ The participants squeezed a dynamometer as hard as possible with each hand twice, with the best measurement of the two attempts selected. This outcome has been used measure previously to evaluate older adults with a hip fracture.^3^ HG has demonstrated high test-retest reliability, with ICC>0.95.^4^

Older adults with hip fracture and caregivers’ health-related quality of life: It was measured through the EuroQol Quality of Life questionnaire (EQ-5D).^5^ The questionnaire consists of two main parts; EQ-5D index, with five dimensions (mobility, self-care, usual activities, pain/discomfort, and anxiety/depression), and EQ-5D VAS, used to evaluate perceived health status from a range of 0 (the worst score) to 100 (the best score).^5^ This outcome measure was already used to assess the quality of life in hip fracture in previous literature.^6^ The internal consistency of the EQ-5D is good with Cronbach’s α=0.83 ^5^ and the test-retest reliability is high with an ICC=0.81.^5^

Functional status: The functional status was assessed using the Functional Independence Measure (FIM).^7^ In the first assessment conducted during the first week after surgery, to fill the FIM, older adults were asked to report their functional status at two different times; i) a week before surgery (i.e., to evaluate an inclusion criterion), and ii) at the moment of the assessment, which was during the week after surgery (i.e., pre-intervention measure). The FIM consists of 18 items, including items related with development of Activities of Daily Living (ADLs) (e.g., self-care or transfers) as well as social and cognitive items (e.g., communication). The total FIM score range is between 18 and 126 points. Higher scores indicate a higher level of independence. The psychometric properties of the FIM scale are adequate.^7^

Older adults with hip fracture and caregivers’ fear of falling**:** We measured it through the Short Falls Efficacy Scale-International (SFES-I), a quick and easy-to-use tool comprised by 7 items relating to some ADLs inside and outside the home. Each item has a score between 1 (not worried about the possibility of fall) and 4 (extremely worried about the possibility of fall). The overall score ranges from 7 (not worried at all about falling) to 28 (extremely worried about falling). The psychometric properties of this scale are adequate.^8^

Pain**:** The numeric rating scale for Pain (NRS) test is a fast way to evaluate the intensity of pain perceived by the patient. The patient indicates the perceived pain by pointing out on a physical scale a value from 0 (without pain) to 10 (maximum pain).^9^ Psychometric properties of the NRS-pain are adequate ^9^.

Older adults with hip fracture and caregivers’ emotional status: It was measured by the Hospital Anxiety and Depression Scale (HADS), which measures the level of anxiety and depression. HADS has been used extensively in the general population.^10^ It is made up of a total of fourteen items with four answers (0–3 points), at the same time divided into two subscales; seven items for anxiety and the other seven for depression. The maximum score for each subscale is twenty-one points. If the score is below 11, it indicates the presence of anxiety or depression. The internal consistency of the HADS is good with Cronbach’s α=0.80.^10^.

Caregivers’ burden: The Caregiver Strain Index is a 13-item tool that caregivers use to indicate whether they are experiencing certain stress-related issues ^11^. Caregivers respond to each item with a simple "Yes" or "No." The total score is determined by adding up the number of "Yes" responses, with a higher score indicating a greater level of stress. This test demonstrated validity, compared to other tools that measure constructs related to burden, and test-retest reliability (ICC = 0.98) in a sample of Spanish women during the puerperium.^12^ Moreover, it also demonstrated good internal consistency (Cronbach’s alpha [α] = 0.83).^13^

Caregivers’ low back pain: We used the Oswestry Disability Index questionnaire^14^ to examine how potential low back pain impacts caregivers' everyday functioning. This tool comprises ten questions, each scored on a scale of 0 to 5. The total score is determined by summing the scores from each section. The Oswestry questionnaire has demonstrated good test-retest reliability (ICC = 0.68) and good internal consistency (α = 0.69).^15^

Caregivers’ fitness self-perception: The International Fitness Scale (IFIS) comprises five questions that inquire about a patient's self-perception of their overall physical fitness, encompassing aspects such as cardiorespiratory, muscular, agility, and flexibility (Ortega et al., 2011). Respondents provide answers on a five-point scale ranging from "very poor" to "very good," with each option assigned a score from 1 to 5. A higher score indicates a more positive perception of physical fitness.^16^ The IFIS has demonstrated validity when compared to objective measures of physical fitness in older adults. ^17^ Furthermore, a recent systematic review with meta-analysis revealed that the IFIS exhibits moderate-to-substantial test-retest reliability (ICC ≥0.60) and good internal consistency (α = 0.888).^18^

References

1. Freire AN, Guerra RO, Alvarado B, et al. Validity and Reliability of the Short Physical Performance Battery in Two Diverse Older Adult Populations in Quebec and Brazil. *J Aging Health* 2012; 24: 863–878.

2. Bohannon RW. Muscle strength. *Curr Opin Clin Nutr Metab Care* 2015; 18: 465–470.

3. Labott BK, Bucht H, Morat M, et al. Effects of Exercise Training on Handgrip Strength in Older Adults: A Meta-Analytical Review. *Gerontology* 2019; 65: 686–698.

4. Ferreira S, Raimundo A, Marmeleira J. Test-retest reliability of the functional reach test and the hand grip strength test in older adults using nursing home services. *Irish J Med Sci (1971 -)* 2021; 190: 1625–1632.

5. Marti C, Hensler S, Herren DB, et al. Measurement properties of the EuroQoL EQ-5D-5L to assess quality of life in patients undergoing carpal tunnel release. *J Hand Surg (European Vol* 2016; 41: 957–962.

6. Amarilla-Donoso FJ, López-Espuela F, Roncero-Martín R, et al. Quality of life in elderly people after a hip fracture: a prospective study. *Health Qual Life Outcomes* 2020; 18: 71.

7. Hobart JC, Lamping DL, Freeman JA, et al. Evidence-based measurement: Which disability scale for neurologic rehabilitation? *Neurology* 2001; 57: 639–644.

8. Kempen GIJMJM, Yardley L, Van Haastregt JCMM, et al. The Short FES-I: a shortened version of the falls efficacy scale-international to assess fear of falling. *Age Ageing* 2007; 37: 45–50.

9. Hawker GA, Mian S, Kendzerska T, et al. Measures of adult pain: Visual Analog Scale for Pain (VAS Pain), Numeric Rating Scale for Pain (NRS Pain), McGill Pain Questionnaire (MPQ), Short-Form McGill Pain Questionnaire (SF-MPQ), Chronic Pain Grade Scale (CPGS), Short Form-36 Bodily Pain Scale (SF. *Arthritis Care Res (Hoboken)* 2011; 63: S240–S252.

10. Bjelland I, Dahl AA, Haug TT, et al. The validity of the Hospital Anxiety and Depression Scale. *J Psychosom Res* 2002; 52: 69–77.

11. Sullivan MT. Caregiver Strain Index (CSI). *J Gerontol Nurs* 2002; 28: 4–5.

12. Feligreras-Alcalá D, Cazalilla-López MDP, Del-Pino-casado R, et al. Validity and reliability of the caregiver strain index scale in women during the puerperium in Spain. *Int J Environ Res Public Health*. Epub ahead of print 2021. DOI: 10.3390/ijerph18073602.

13. Kruithof WJ, Post MWMM, Visser-Meily JMA. Measuring negative and positive caregiving experiences: A psychometric analysis of the Caregiver Strain Index Expanded. *Clin Rehabil*. Epub ahead of print 2015. DOI: 10.1177/0269215515570378.

14. Fairbank JCT, Pynsent PB. The oswestry disability index. *Spine (Phila Pa 1976)*. Epub ahead of print 2000. DOI: 10.1097/00007632-200011150-00017.

15. Baradaran A, Ebrahimzadeh MH, Birjandinejad A, et al. Cross-cultural adaptation, validation, and reliability testing of the modified oswestry disability questionnaire in persian population with low back pain. *Asian Spine J*. Epub ahead of print 2016. DOI: 10.4184/asj.2016.10.2.215.

16. Ortega FB, Ruiz JR, España-Romero V, et al. The International Fitness Scale (IFIS): Usefulness of self-reported fitness in youth. *Int J Epidemiol*. Epub ahead of print 2011. DOI: 10.1093/ije/dyr039.

17. Merellano-Navarro E, Collado-Mateo D, García-Rubio J, et al. Validity of the International Fitness Scale “IFIS” in older adults. *Exp Gerontol*. Epub ahead of print 2017. DOI: 10.1016/j.exger.2017.05.001.

18. Pereira D de A, Correia Júnior JL, Carvas Junior N, et al. Reliability of questionnaire The International Fitness Scale: a systematic review and meta-analysis. *Einstein (Sao Paulo)*. Epub ahead of print 2020. DOI: 10.31744/einstein_journal/2020rw5232.

**Appendix 2.** Normality assessments


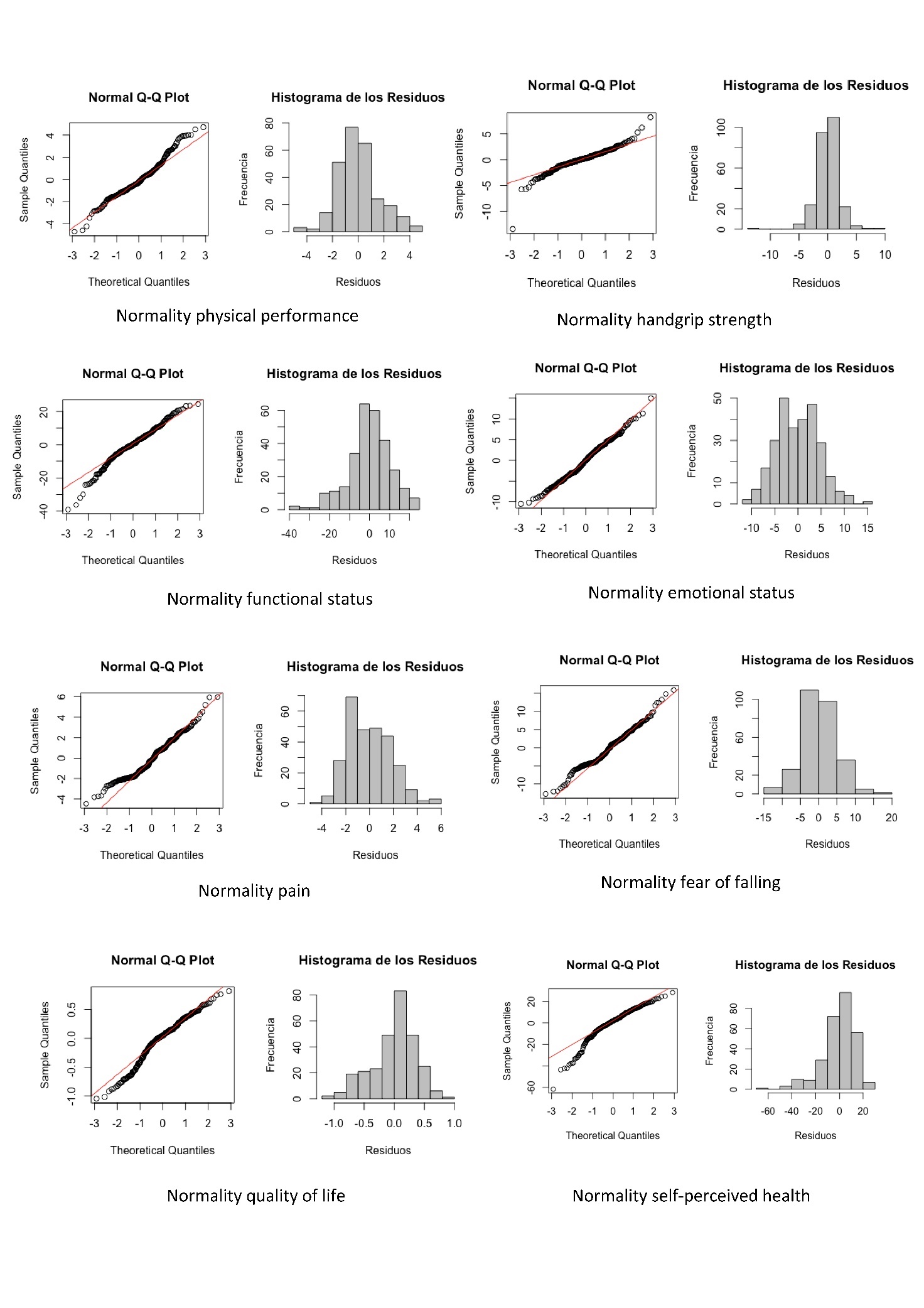


**Figure S1.** Q-Q plots and histograms to explore normality of residuals of patients outcomes


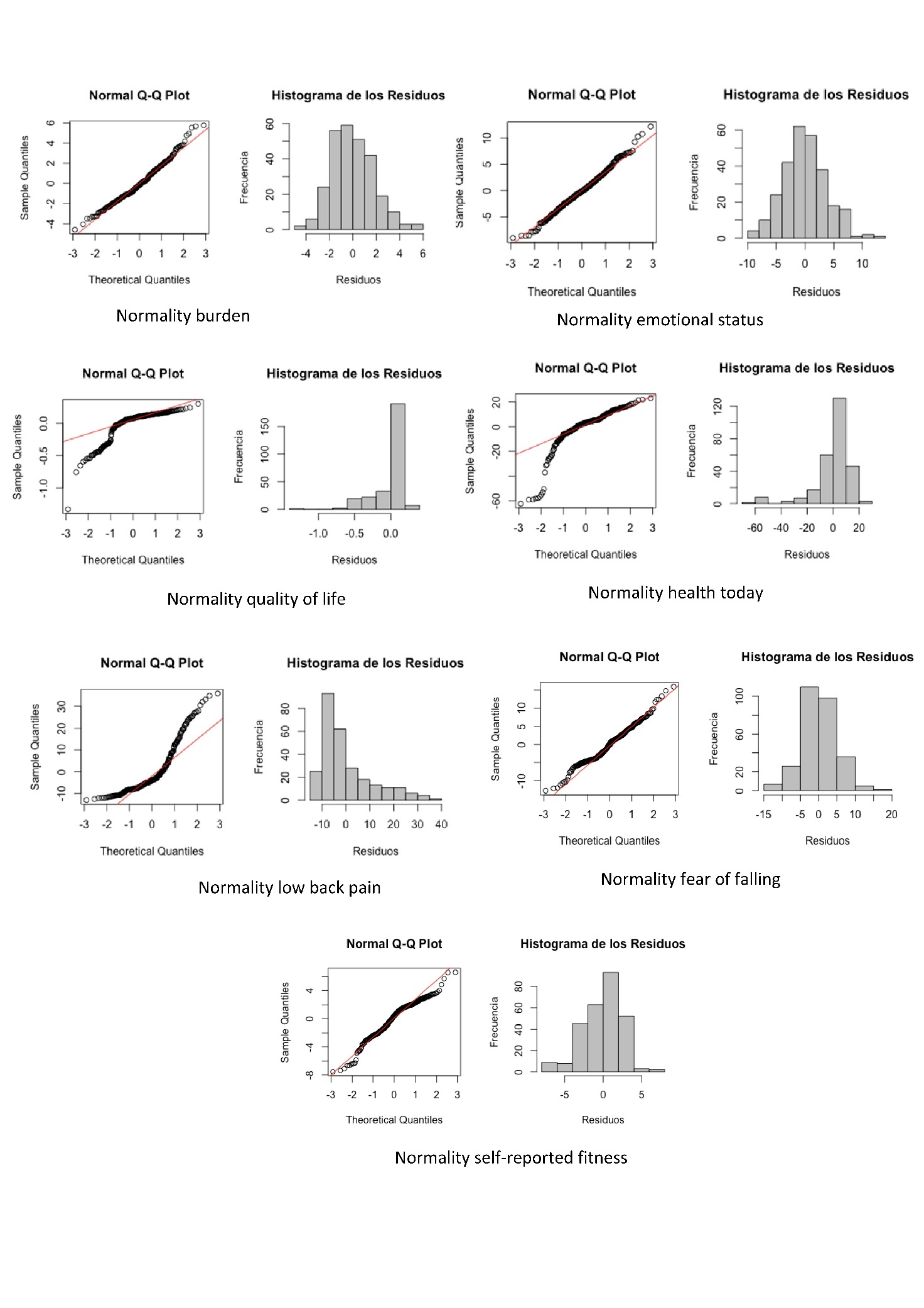


**Figure S2.** Q-Q plots and histograms to explore normality of residuals of caregivers outcomes

# Appendix 3: Results of per-protocol analyses (Meeting criteria of activity) and sensitivity analyses.


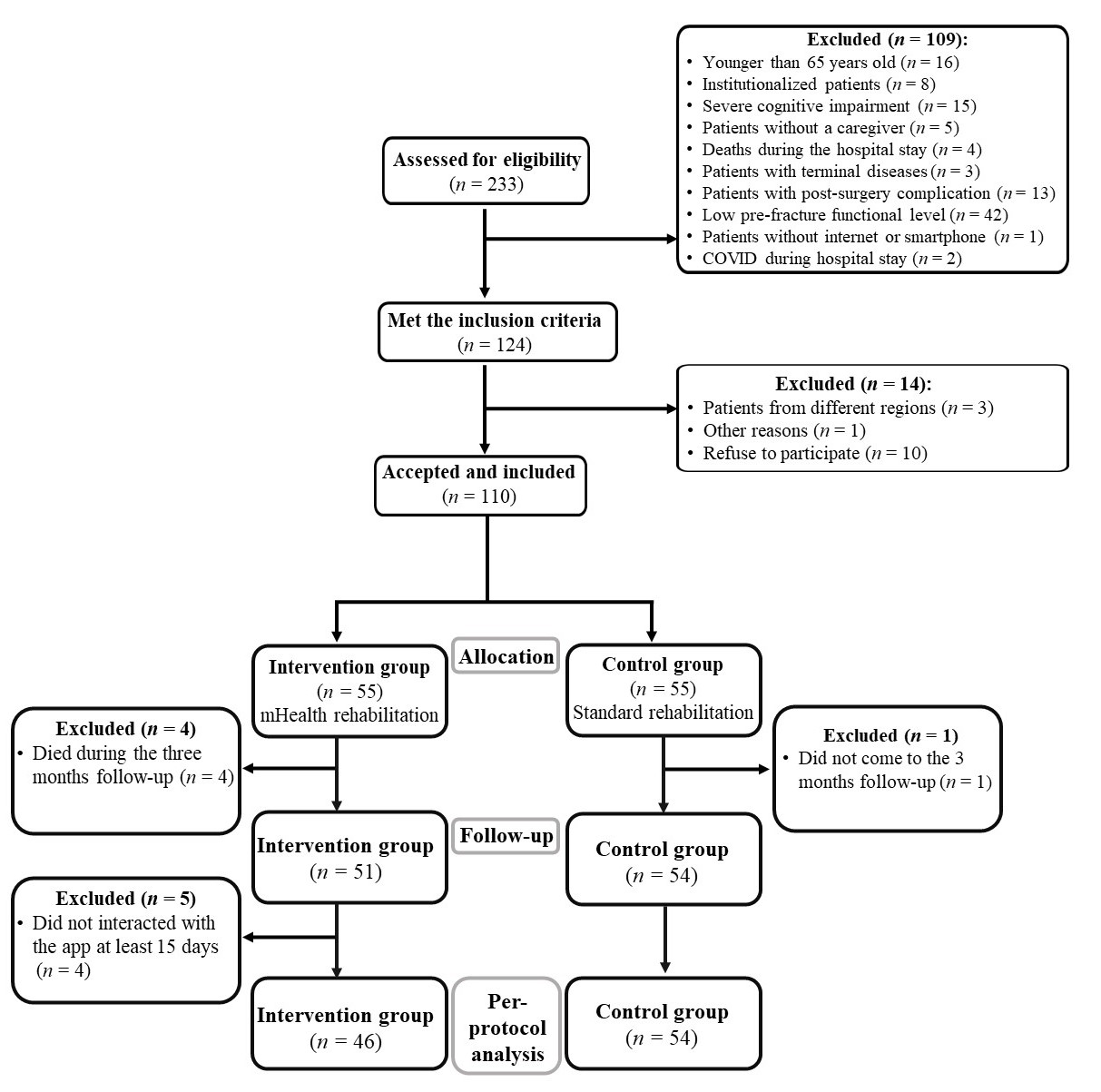


**Figure S3.** Consort flowchart of the sample recruited for this study.

| **Variable**  **Table S1.** Baseline characteristics of participants included in per-protocol analysis | **Intervention group (*n*= 46)** | **Control group (*n*= 54)** |
| --- | --- | --- |
| Age (years), Mean (SD) | 79·70 (7·15) | 79·94 (7·74) |
| Sex, *n* (%) | |  |
| Women | 33 (72) | 38 (70) |
| Men | 13 (28) | 16 (30) |
| Type of injury, *n* (%) |  |  |
| Fracture Cervical Femoral (Intracapsular) | 26 (57) | 30 (56) |
| Fracture Trochanteric (Extracapsular) | 20 (43) | 24 (44) |
| Type of surgery, *n* (%) |  |  |
| Prosthesis | 14 (65) | 13 (24) |
| Screw Plate | 30 (31) | 36 (67) |
| PFN-A Nail | 2 (4) | 5 (9) |
| Falls in the previous year, *n* (%) |  |  |
| Yes | 16 (35) | 18 (33) |
| No | 30 (65) | 36 (67) |
| Pre-fracture residence, *n* (%) |  |  |
| Own home | 45 (98) | 54 (100) |
| Nursing or relative´s home | 1 (2) | 0 (0) |
| Post-fracture residence, *n* (%) |  |  |
| Own home | 42 (91) | 47 (87) |
| Nursing or relative´s home | 4 (9) | 7 (13) |
| Hospital stay (days), Mean (SD) | 6·83 (4·2) | 5·64 (3·3) |
| **Older adults’ outcomes** | | |
| Objectively measured physical performance (SPPB 0-12) | 2·67 (0·86) | 2·63 (1·22) |
| Functional status (FIM, 18-126) | 76·082 (17·52) | 80·47 (14·40) |
| Emotional status (HADS, 0-42) | 15·30 (4·92) | 15·04 (5·78) |
| Pain level (NRS, 0-10) | 6·23 (2·10) | 6·52 (1·78) |
| Fear of falling (SFES-I, 7-28) | 19·70 (7·33) | 20·016 (5·15) |
| Quality of life (EQ5D -0·65-1) | 0·30 (0·28) | 0·35 (0·49) |
| **Family caregivers’ outcomes** |  |  |
| Caregivers burden (CSI, 0-13) | 6·30 (1·94) | 6·72 (1·83) |
| Emotional status (HADS, 0-42) | 12·43 (4·013) | 12·90 (3·59) |
| Low back pain (ODI, 0-50) | 10·13 (12·97) | 9·00 (12·56) |
| Quality of life (EQ5D, -0·65-1) | 0·82 (0·29) | 0·85 (0·21) |
| Fear of falling (SFES-I, 7-28) | 20·5 (6·56) | 20·67 (5·72) |
| Self-reported fitness (IFIS, 4-20) | 17·14 (3·32) | 17·02 (3·24) |
| Values are Mean (SD) unless otherwise indicated. CSI: Caregivers’ Strain Index; EQ5D: EuroQol-5D; FIM= Functional Independence Measure; HADS: Hospital Anxiety and Depression Scale; IFIS: International Fitness Scale; NRS= Numeric rating scale; ODI: Oswestry Low Back Disability; PFN-A= Proximal Femoral Nail; SD= standard deviation; SFES-I= Short Falls Efficacy Scale; SPPB: Short Physical Performance Battery. | | |

| **Table S2.** Differences in older adults’ outcomes between groups at 3-month after surgery (post intervention) and at 1-year follow-up: per-protocol analyses | | | | | | | | | | | |
| --- | --- | --- | --- | --- | --- | --- | --- | --- | --- | --- | --- |
|  |  | Intervention group (ActiveHip+ mHealth) | | |  | Control group (Usual care) | | |  |  |  |
| Outcome | Month | N | Mean (SE) | Change from  Baseline, Mean (SE) |  | n | Mean (SE) | Change from  Baseline, Mean (SE) | Differences in change from baseline  (ActiveHip+ vs. Usual care), Mean (SE) | *p*_uncorrected_ | *p*_FDR_ |
| Objectively measured physical performance (SPPB, 0-12) | 0 | 46 | 2·67 (0·12) | Reference |  | 54 | 2·67 (0·12) | Reference | Reference | Reference | Reference |
|  | 3 | 46 | 7·15 (0·34) | 4·48 (0·35) |  | 54 | 5·71 (0·31) | 3·04 (0·33) | -1·44 (0·36) | **<0·0001** | **0·0019** |
|  | 12 | 28 | 6·48 (0·35) | 3·81 (0·36) |  | 29 | 6·17 (0·34) | 3·50 (0·35) | -0·34 (0·48) | 0·48 | 0·99 |
| Balance (SPPB, 0–4) | 0 | 46 | 1·48 (0·06) | Reference |  | 54 | 1·48 (0·06) | Reference | Reference | Reference | Reference |
|  | 3 | 46 | 3·59 (0·10) | 2·11 (0·13) |  | 54 | 3·18 (0·09) | 1·70 (0·12) | -0·40 (0·16) | **0·015** | **0·041** |
|  | 12 | 28 | 4·12 (0·19) | 2·64 (0·20) |  | 29 | 3·91 (0·18) | 2·44 (0·19) | -0·18 (0·20) | 0·36 | 0·99 |
| Gait speed (SPPB, 0–4) | 0 | 46 | 0·67 (0·05) | Reference |  | 54 | 0·67 (0·05) | Reference | Reference | Reference | Reference |
|  | 3 | 46 | 1·78 (0·14) | 1·11 (0·15) |  | 54 | 1·26 (0·13) | 0·58 (0·14) | -0·51 (0·14) | **0·0034** | **0·016** |
|  | 12 | 28 | 1·21 (0·09) | 0·54 (0·09) |  | 29 | 1·27 (0·05) | 0·59 (0·09) | 0·03 (0·18) | 0·87 | 0·99 |
| Chair stand (SPPB, 0–4) | 0 | 46 | 0·39 (0·05) | Reference |  | 54 | 0·39 (0·05) | Reference | Reference | Reference | Reference |
|  | 3 | 46 | 1·79 (0·14) | 1·40 (0·15) |  | 54 | 1·26 (0·13) | 0·86 (0·14) | -0·60 (0·23) | **0·0018** | **0·011** |
|  | 12 | 28 | 1·93 (0·20) | 1·54 (0·21) |  | 29 | 1·74 (0·20) | 1·35 (0·21) | -0·35 (0·27) | 0·21 | 0·99 |
| Handgrip strength: Kg | 0 | 46 | 18·10 (0·52) | Reference |  | 54 | 18·10 (0·52) | Reference | Reference | Reference | Reference |
|  | 3 | 44 | 20·60 (0·48) | 2·44 (0·50) |  | 52 | 19·90 (0·46) | 1·74 (0·47) | -0·74 (0·68) | 0·28 | 0·38 |
|  | 12 | 24 | 20·40 (0·62) | 2·24 (0·63) |  | 28 | 19·60 (0·59) | 1·47 (0·59) | -0·94 (0·88) | 0·29 | 0·99 |
| Functional status (FIM, 18-126) | 0 | 46 | 76·80 (1·54) | Reference |  | 54 | 76·80 (1·54) | Reference | Reference | Reference | Reference |
|  | 3 | 46 | 114·90 (1·41) | 38·13 (1·82) |  | 54 | 110·70 (1·31) | 33·91 (1·74) | 37·99 (2·18) | 0·14 | 0·24 |
|  | 12 | 32 | 108·40 (2·80) | 31·63 (3·06) |  | 37 | 105·80 (2·59) | 28·95 (2·87) | 32·95 (2·52) | 0·28 | 0·99 |
| FIM self-care (6–42) | 0 | 46 | 19·00 (0·56) | Reference |  | 54 | 19·00 (0·56) | Reference | Reference | Reference | Reference |
|  | 3 | 46 | 37·10 (0·73) | 18·12 (0·85) |  | 54 | 34·50 (0·68) | 15·58 (0·80) | -2·49 (1·12) | **0·026** | 0·062 |
|  | 12 | 32 | 35·00 (1·18) | 15·99 (1·26) |  | 37 | 33·50 (1·10) | 14·59 (1·18) | -1·42 (1·12) | 0·29 | 0·99 |
| FIM sphincter (2–14) | 0 | 46 | 12·20 (0·25) | Reference |  | 54 | 12·20 (0·25) | Reference | Reference | Reference | Reference |
|  | 3 | 46 | 12·40 (0·14) | 2·23 (0·28) |  | 54 | 12·90 (0·13) | 1·68 (0·27) | -0·54 (0·37) | 0·14 | 0·24 |
|  | 12 | 32 | 13·00 (0·30) | 1·73 (0·39) |  | 37 | 12·20 (0·28) | 0·95 (0·38) | -0·77 (0·44) | 0·082 | 0·95 |
| FIM transfer (3-21) | 0 | 46 | 8·13 (0·56) | Reference |  | 54 | 8·13 (0·56) | Reference | Reference | Reference | Reference |
|  | 3 | 46 | 18·40 (0·34) | 10·26 (0·59) |  | 54 | 17·77 (0·31) | 9·64 (0·57) | -0·41 (0·80) | 0·61 | 0·68 |
|  | 12 | 32 | 17·15 (0·62) | 9·02 (0·80) |  | 37 | 17·19 (0·57) | 9·06 (0·77) | -0·13 (0·97) | 0·89 | 0·99 |
| FIM locomotion (2-14) | 0 | 46 | 5·10 (0·34) | Reference |  | 54 | 5.10 (0.34) | Reference | Reference | Reference | Reference |
|  | 3 | 46 | 12·00 (0·24) | 6.90 (0.39) |  | 54 | 11.50 (0.22) | 6.40 (0.37) | -0.46 (0.53) | 0.38 | 0·48 |
|  | 12 | 32 | 5·10 (0·34) | 6.01 (0.55) |  | 37 | 11.50 (0.43) | 6.42 (0.53) | 0.15 (0.63) | 0.81 | 0·99 |
| FIM communication (2-14) | 0 | 46 | 13·50 (0·10) | Reference |  | 54 | 13.50 (0.10) | Reference | Reference | Reference | Reference |
|  | 3 | 46 | 13·90 (0·09) | 0.35 (0.13) |  | 54 | 13.70 (0.08) | 0.19 (0.13) | -0.12 (0.20) | 0.54 | 0·64 |
|  | 12 | 32 | 13·40 (0·24) | -0.13 (0.25) |  | 37 | 12.90 (0.22) | -0.67 (0.24) | -0.53 (0.24) | **0.023** | 0·95 |
| FIM psychosocial (3-21) | 0 | 46 | 19·90 (0·20) | Reference |  | 54 | 19.90 (0.20) | Reference | Reference | Reference | Reference |
|  | 3 | 46 | 20·20 (0·20) | 0.35 (0.28) |  | 54 | 20.20 (0.20) | 0.34 (0.27) | 0.03 (0.39) | 0.93 | 0·97 |
|  | 12 | 32 | 19·40 (0·44) | -0.45 (0.46) |  | 37 | 18.80 (0.41) | -1.05 (0.43) | -0.73 (0.46) | 0.12 | 0·95 |
| Emotional status (HADS, 0-42) | 0 | 46 | 15·30 (0·58) | Reference |  | 54 | 15.30 (0.58) | Reference | Reference | Reference | Reference |
|  | 3 | 46 | 11·50 (0·69) | -3·88 (0·80) |  | 54 | 14·10 (0·63) | -1·25 (0·76) | 2·57 (1·01) | **0·011** | **0·035** |
|  | 12 | 31 | 15·50 (0·80) | 0·15 (0·97) |  | 37 | 15·44 (0·73) | 0·05 (0·92) | -0·31 (1·22) | 0·80 | 0·99 |
| HADS Anxiety (0-21) | 0 | 46 | 7·67 (0·41) | Reference |  | 54 | 7·67 (0·41) | Reference | Reference | Reference | Reference |
|  | 3 | 46 | 3·94 (0·51) | -3·73 (0·58) |  | 54 | 6·51 (0·47) | -1·16 (0·55) | 2·52 (0·73) | **0·00068** | **0·0065** |
|  | 12 | 31 | 5·76 (0·57) | -1·92 (0·69) |  | 37 | 5·89 (0·52) | -1·78 (0·65) | -0·07 (0·89) | 0·94 | 0·99 |
| HADS Depression (0-21) | 0 | 46 | 7·66 (0·25) | Reference |  | 54 | 7·66 (0·25) | Reference | Reference | Reference | Reference |
|  | 3 | 46 | 7·52 (0·34) | -0·14 (0·38) |  | 54 | 7·55 (0·31) | -0·11 (0·35) | 0·02 (0·47) | 0·97 | 0·97 |
|  | 12 | 31 | 9·73 (0·41) | 2·07 (0·48) |  | 37 | 9·52 (0·38) | 1·86 (0·45) | -0·18 (0·57) | 0·76 | 0·99 |
| Pain (NRS, 0-10) | 0 | 46 | 6·23 (0·20) | Reference |  | 54 | 6·23 (0·20) | Reference | Reference | Reference | Reference |
|  | 3 | 46 | 1·21 (0·29) | -5·02 (0·36) |  | 54 | 2·16 (0·27) | -4·07 (0·34) | 0·88 (0·41) | **0·031** | 0·065 |
|  | 12 | 32 | 1·78 (0·40) | -4·45 (0·43) |  | 37 | 2·14 (0·34) | -4·09 (0·40) | 0·40 (0·49) | 0·42 | 0·99 |
| Fear of Falling (SFES-I, 7-28) | 0 | 46 | 19·70 (0·62) | Reference |  | 54 | 19·70 (0·62) | Reference | Reference | Reference | Reference |
|  | 3 | 46 | 11·70 (0·80) | -8·01 (0·97) |  | 54 | 13·30 (0·74) | -6·46 (0·92) | 1·50 (1·13) | 0·19 | 0·30 |
|  | 12 | 32 | 13·40 (0·97) | -6·35 (1·09) |  | 37 | 13·10 (0·89) | -6·61 (1·02) | -0·22 (1·36) | 0·87 | 0·99 |
| Quality of Life (EQ5D, -0.65–1) | 0 | 46 | 0·30 (0·05) | Reference |  | 54 | 0·30 (0·05) | Reference | Reference | Reference | Reference |
|  | 3 | 46 | 0·41 (0·07) | 0·19 (0·07) |  | 54 | 0·32 (0·06) | 0·01 (0·07) | -0·10 (0·09) | 0·28 | 0·38 |
|  | 12 | 32 | 0·47 (0·08) | 0·16 (0·08) |  | 37 | 0·54 (0·07) | 0·24 (0·08) | 0·07 (0·11) | 0·50 | 0·99 |
| Health Today (EQ5D, 0-100) | 0 | 46 | 54·70 (2·25) | Reference |  | 54 | 54·70 (2·25) | Reference | Reference | Reference | Reference |
|  | 3 | 46 | 79·90 (2·25) | 25·18 (2·79) |  | 54 | 72·10 (2·08) | 17·40 (2·65) | -9·15 (3·57) | **0·011** | **0·035** |
|  | 12 | 32 | 73·50 (3·00) | 18·80 (3·37) |  | 37 | 69·80 (2·80) | 15·06 (3·19) | -4·49 (4·25) | 0·29 | 0·99 |
| EQ5D: EuroQol-5D; FDR: False Discovery Rate; FIM= Functional Independence Measure; HADS: Hospital Anxiety and Depression Scale; HG: Handgrip; n=sample size; NRS= Numeric rating scale; SE: Standard Error SFES-I= Short Falls Efficacy Scale; SPPB: Short Physical Performance Battery. Significant differences (p < 0·05) are highlighted in bold. | | | | | | | | | | | |


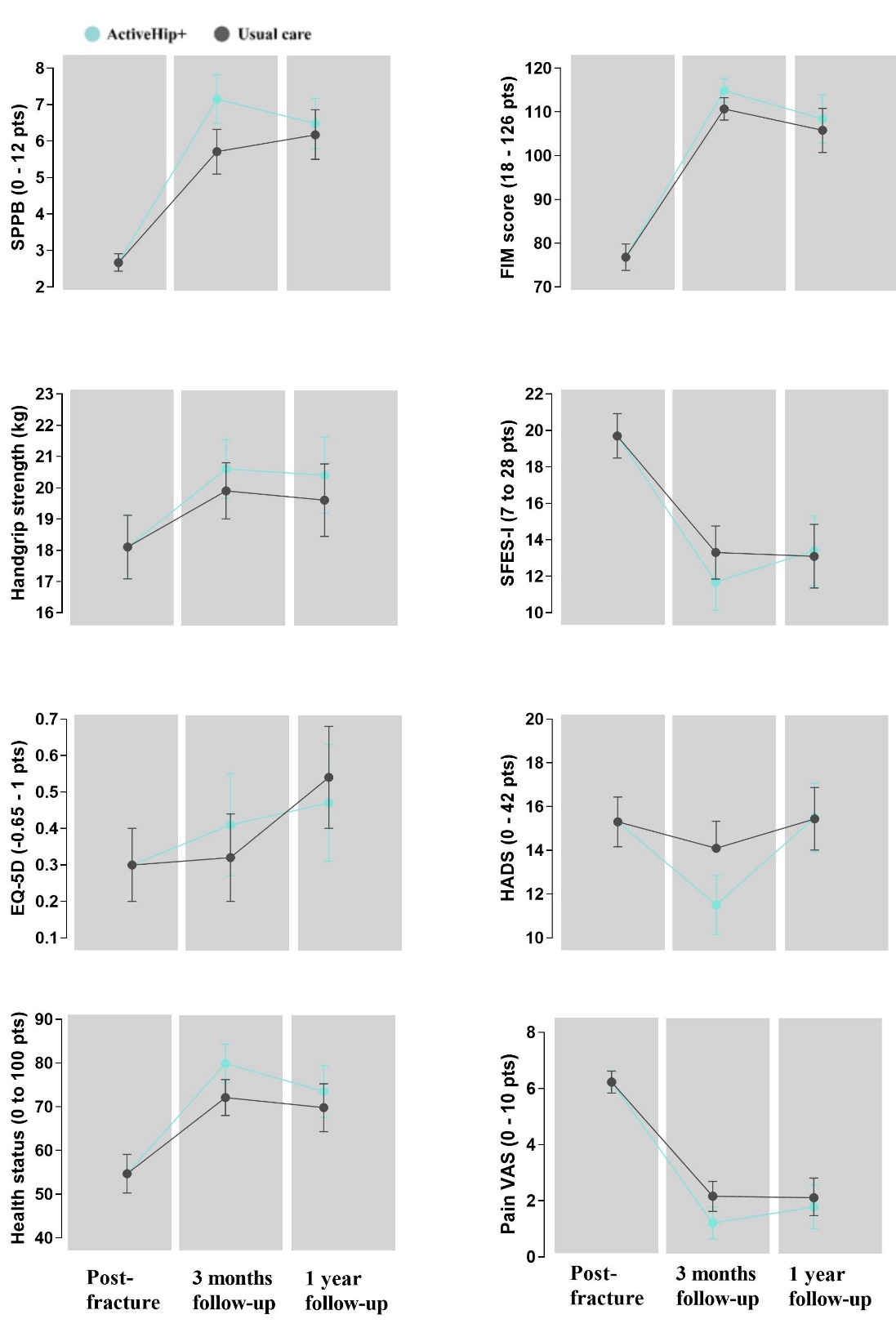
**Figure S4.** Changes in older adults' outcomes by time and group: per-protocol analyses. Data points represent the model-estimated means and 95% confidence intervals (indicated by the I bars) from a constrained linear mixed model (cLMM) with baseline means constrained to be equal across study arms, reflecting the pre-randomisation nature of the baseline assessment.

| **Table 3.** Differences in family caregivers' outcomes between groups at 3-month after surgery (post intervention) and at 1-year follow-up: per-protocol analyses | | | | | | | | | | | |
| --- | --- | --- | --- | --- | --- | --- | --- | --- | --- | --- | --- |
|  |  | Intervention group (ActiveHip+ mHealth) | | |  | Control group (Usual care) | | |  |  |  |
| Outcome | Months | n | Mean (SE) | Change from  Baseline, Mean (SE) |  | n | Mean (SE) | Change from  Baseline, Mean (SE) | Differences in change from baseline  (ActiveHip+ vs. Usual care),  Mean (SE) | *p*_uncorrected_ | *p*_FDR_ |
| Care Strain (CSI, 0-13) | 0 | 46 | 6·30 (0·19) | Reference |  | 54 | 6·30 (0·19) | Reference | Reference | Reference | Reference |
|  | 3 | 46 | 3·65 (0·35) | -2·65 (0·37) |  | 54 | 4·58 (0·33) | -1·72 (0·35) | 0·93 (0·47) | **0·046** | 0·21 |
|  | 12 | 30 | 3·75 (0·56) | -2·55 (0·58) |  | 31 | 3·91 (0·54) | -2·39 (0·56) | -0·04 (0·59) | 0·95 | 0·95 |
| Emotional status (HADS,0–42) | 0 | 46 | 12·43 (0·44) | Reference |  | 54 | 12·43 (0·44) | Reference | Reference | Reference | Reference |
|  | 3 | 46 | 9·07 (0·56) | -3·36 (0·71) |  | 54 | 10·62 (0·52) | -1·81 (0·68) | 1·45 (0·80) | 0·07 | 0·21 |
|  | 12 | 30 | 16·04 (0·68) | 3·61 (0·79) |  | 31 | 15·32 (0·66) | 2·89 (0·78) | -0·65 (1·02) | 0·52 | 0·89 |
| HADS anxiety (0-21) | 0 | 46 | 3·91 (0·32) | Reference |  | 54 | 3·91 (0·32) | Reference | Reference | Reference | Reference |
|  | 3 | 46 | 1·87 (0·35) | -2·04 (0·48) |  | 54 | 2·44 (0·32) | -1·47 (0·46) | 0·46 (0·54) | 0·40 | 0·61 |
|  | 12 | 30 | 7·09 (0·43) | 3·18 (0·53) |  | 31 | 6·97 (0·42) | 3·06 (0·52) | -0·05 (0·69) | 0·94 | 0·95 |
| HADS depression (0-21) | 0 | 46 | 8·52 (0·34) | Reference |  | 54 | 8·52 (0·34) | Reference | Reference | Reference | Reference |
|  | 3 | 46 | 7·20 (0·34) | -1·32 (0·39) |  | 54 | 8·18 (0·32) | -0·34 (0·37) | 0·99 (0·42) | **0·020** | 0·18 |
|  | 12 | 30 | 8·92 (0·34) | 0·40 (0·38) |  | 31 | 8·30 (0·33) | -0·22 (0·38) | -0·61 (0·54) | 0·26 | 0·89 |
| Quality of Life (EQ5D, -0.65–1) | 0 | 46 | 0·82 (0·03) | Reference |  | 54 | 0·82 (0·03) | Reference | Reference | Reference | Reference |
|  | 3 | 46 | 0·92 (0·03) | 0·10 (0·04) |  | 54 | 0·86 (0·03) | 0·04 (0·04) | -0·06 (0·05) | 0·24 | 0·54 |
|  | 12 | 30 | 0·85 (0·04) | 0·03 (0·05) |  | 31 | 0·87 (0·04) | 0·05 (0·05) | 0·03 (0·06) | 0·65 | 0·89 |
| Health Today (EQ5D,0–100) | 0 | 46 | 73·50 (2·25) | Reference |  | 54 | 73·50 (2·25) | Reference | Reference | Reference | Reference |
|  | 3 | 46 | 84·90 (1·81) | 11·40 (2·48) |  | 54 | 84·20 (1·43) | 10·69 (2·41) | -0·84 (3·21) | 0·79 | 0·89 |
|  | 12 | 30 | 73·50 (2·25) | 11·47 (2·85) |  | 31 | 82·60 (1·75) | 9·08 (2·81) | -3·29 (4·08) | 0·42 | 0·89 |
| Low back pain (ODI,0–50) | 0 | 46 | 10·13 (1·35) | Reference |  | 54 | 10·13 (1·35) | Reference | Reference | Reference | Reference |
|  | 3 | 46 | 4·78 (1·48) | -5·35 (1·97) |  | 54 | 6·63 (1·36) | -3·50 (1·88) | 1·99 (2·41) | 0·41 | 0·61 |
|  | 12 | 30 | 10·35 (2·43) | 0·21 (2·73) |  | 31 | 13·53 (2·36) | 3·39 (2·67) | 3·81 (3·07) | 0·22 | 0·89 |
| Fear of Falling (SFES-I, 7–28) | 0 | 46 | 20·50 (0·61) | Reference |  | 54 | 20·50 (0·61) | Reference | Reference | Reference | Reference |
|  | 3 | 46 | 11·20 (0·95) | -9·29 (0·94) |  | 54 | 11·90 (0·71) | -8·64 (0·89) | 0·61 (1·09) | 0·58 | 0·75 |
|  | 12 | 30 | 12·20 (0·95) | -8·34 (0·89) |  | 31 | 11·30 (0·91) | -9·23 (1·01) | -0·56 (1·39) | 0·69 | 0·89 |
| Self-reported Fitness (IFIS, 4–20) | 0 | 46 | 17·10 (0·33) | Reference |  | 54 | 17·10 (0·33) | Reference | Reference | Reference | Reference |
|  | 3 | 46 | 17·50 (0·43) | 0·44 (0·50) |  | 54 | 17·50 (0·40) | 0·42 (0·47) | -0·01 (0·61) | 0·98 | 0·98 |
|  | 12 | 30 | 17·20 (0·50) | 0·07 (0·60) |  | 31 | 16·40 (0·50) | -0·69 (0·60) | -0·68 (0·76) | 0·37 | 0·89 |
| CSI: Caregivers’ Strain Index; EQ5D: EuroQol-5D; FDR: False Discovery Rate; HADS: Hospital Anxiety and Depression Scale; IFIS: International Fitness Scale; n=sample size; ODI: Oswestry Low Back Disability; SE: Standard Error; SFES-I= Short Falls Efficacy Scale. Significant differences (p < 0·05) are highlighted in bold. | | | | | | | | | | | |


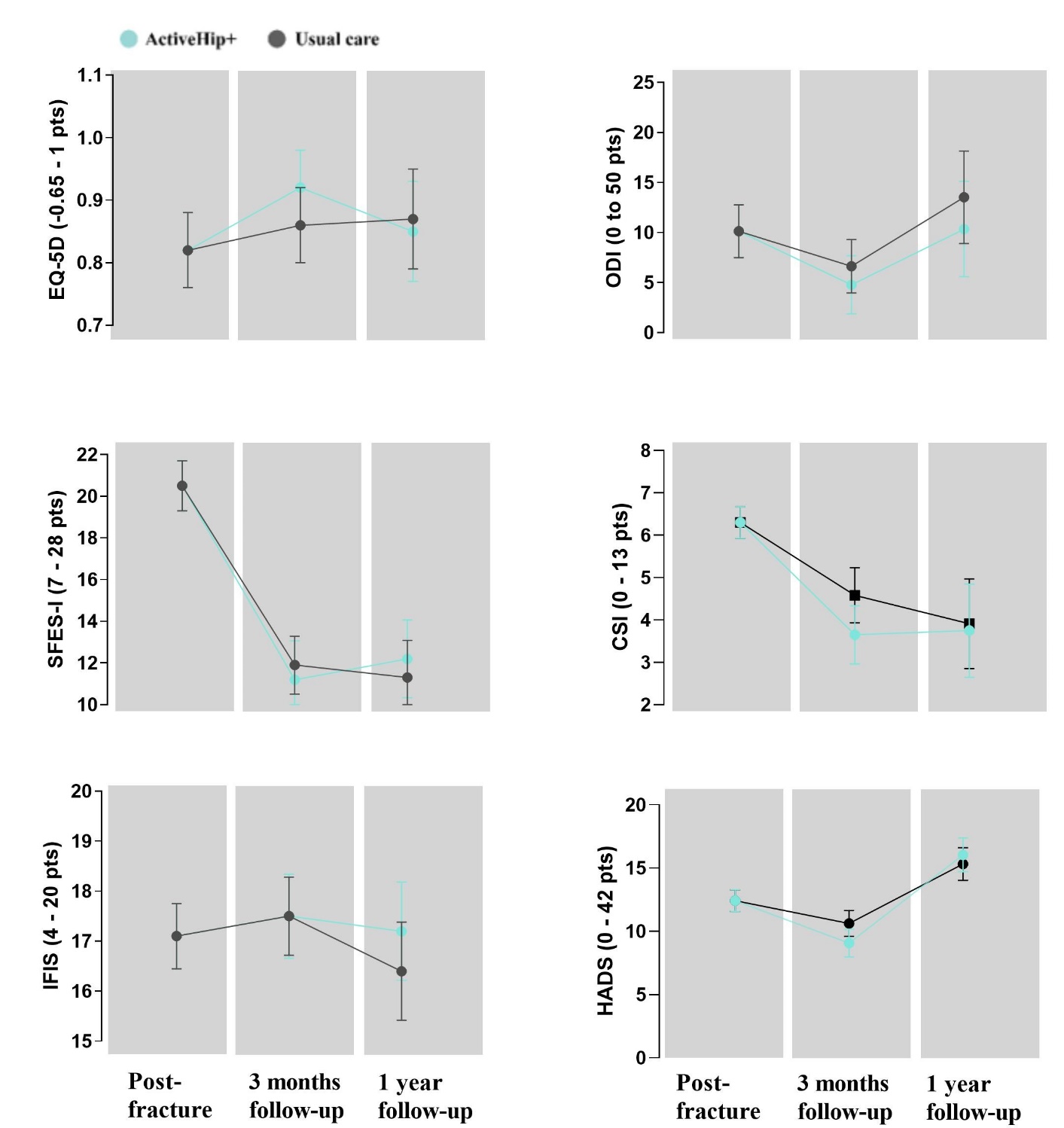


**Figure S5.** Changes in family caregivers' outcomes by time and group: per-protocol analyses. Data points represent the model-estimated means and 95% confidence intervals (indicated by the I bars) from a constrained linear mixed model (cLMM) with baseline means constrained to be equal across study arms, reflecting the pre-randomisation nature of the baseline assessment.

| **Table S4.** Differences in older adults’ outcomes between groups at 3-month after surgery (post intervention) and at 1-year follow-up: sensitivity analyses. | | | | | | | | | | | |
| --- | --- | --- | --- | --- | --- | --- | --- | --- | --- | --- | --- |
|  |  | Intervention group (ActiveHip+ mHealth) | | |  | Control group (Usual care) | | |  |  |  |
| Outcome | Month | n | Mean (SE) | Change from  Baseline, Mean (SE) |  | n | Mean (SE) | Change from  Baseline, Mean (SE) | Differences in change from baseline  (ActiveHip+ minus Usual care), Mean (SE) | *p*_uncorrected_ | *p*_FDR_ |
| Objectively measured  physical performance  (SPPB, 0 – 12) | 0 | 51 | 2·69 (0·12) | Ref |  | 54 | 2·69 (0·12) | Ref | Ref | Ref | Ref |
|  | 3 | 51 | 7·11 (0·32) | 4·43 (0·34) |  | 54 | 5·71 (0·32) | 3·02 (0·33) | 1·40 (0·35) | **0·000081** | **0·0015** |
|  | 12 | 51 | 6·32 (0·24) | 3·63 (0·27) |  | 54 | 6·18 (0·24) | 3·50 (0·26) | 0·12 (0·35) | 0·74 | 0·83 |
| Balance  (SPPB, 0–4) | 0 | 51 | 1·48 (0·06) | Ref |  | 54 | 1·48 (0·06) | Ref | Ref | Ref | Ref |
|  | 3 | 51 | 3·59 (0·10) | 2·11 (0·12) |  | 54 | 3·18 (0·10) | 1·71 (0·12) | 0·40 (0·16) | **0·015** | **0·048** |
|  | 12 | 51 | 4·00 (0·15) | 2·53 (0·17) |  | 54 | 3·91 (0·15) | 2·43 (0·16) | 0·09 (0·16) | 0·58 | 0·83 |
| Gait Speed  (SPPB 0 – 4) | 0 | 51 | 0·68 (0·05) | Ref |  | 54 | 0·68 (0·05) | Ref | Ref | Ref | Ref |
|  | 3 | 51 | 1·80 (0·14) | 1·08 (0·14) |  | 54 | 1·26 (0·13) | 0·59 (0·14) | 0·50 (0·14) | **0·00029** | **0·0028** |
|  | 12 | 51 | 1·31 (0·08) | 0·63 (0·09) |  | 54 | 1·41 (0·07) | 0·74 (0·08) | -0·10 (0·14) | 0·48 | 0·83 |
| Chair Stand  (SPPB, 0 – 4) | 0 | 51 | 0·41 (0·05) | Ref |  | 54 | 0·41 (0·05) | Ref | Ref | Ref | Ref |
|  | 3 | 51 | 1·77 (0·14) | 1·36 (0·14) |  | 54 | 1·26 (0·13) | 0·85 (0·14) | 0·51 (0·17) | **0·0029** | **0·014** |
|  | 12 | 51 | 2·00 (0·14) | 1·59 (0·15) |  | 54 | 1·87 (0·14) | 1·46 (0·15) | 0·13 (0·17) | 0·44 | 0·83 |
| Handgrip strength  Kg | 0 | 51 | 18·2 (0·51) | Ref |  | 54 | 18·2 (0·51) | Ref | Ref | Ref | Ref |
|  | 3 | 51 | 20·5 (0·47) | 2·35 (0·48) |  | 54 | 19·7 (0·46) | 1·58 (0·47) | 0·77 (0·95) | 0·42 | 0·53 |
|  | 12 | 51 | 18·9 (0·85) | 0·73 (0·94) |  | 54 | 19·7 (0·83) | 1·57 (0·92) | -0·83 (0·95) | 0·38 | 0·83 |
| Functional status  (FIM, 18-126) | 0 | 51 | 77·3 (1·51) | Ref |  | 54 | 77·3 (1·51) | Ref | Ref | Ref | Ref |
|  | 3 | 51 | 114·5 (1·34) | 37·20 (1·76) |  | 54 | 110·8 (1·30) | 33·50 (1·73) | 3·41 (2·53) | 0·18 | 0·34 |
|  | 12 | 51 | 109·0 (2·07) | 31·66 (2·44) |  | 54 | 106·2 (2·02) | 28·86 (2·39) | 2·26 (2·53) | 0·37 | 0·83 |
| FIM self-care  (6–42) | 0 | 51 | 19·0 (0·55) | Ref |  | 54 | 19·0 (0·55) | Ref | Ref | Ref | Ref |
|  | 3 | 51 | 36·8 (0·70) | 17·74 (0·81) |  | 54 | 34·6 (0·68) | 15·53 (0·80) | 2·19 (1·08) | **0·043** | 0·10 |
|  | 12 | 51 | 35·2 (0·89) | 16·18 (0·99) |  | 54 | 34·7 (0·87) | 15·66 (0·96) | 0·48 (1·08) | 0·66 | 0·83 |
| FIM sphincter  (2–14) | 0 | 51 | 11·3 (0·24) | Ref |  | 54 | 11·3 (0·24) | Ref | Ref | Ref | Ref |
|  | 3 | 51 | 13·3 (0·14) | 2·08 (0·27) |  | 54 | 12·9 (0·14) | 1·64 (0·27) | 0·45 (0·35) | 0·21 | 0·36 |
|  | 12 | 51 | 12·8 (0·23) | 1·54 (0·34) |  | 54 | 12·2 (0·23) | 0·99 (0·33) | 0·58 (0·35) | 0·10 | 0·48 |
| FIM transfer  (3-21) | 0 | 51 | 8·4 (0·56) | Ref |  | 54 | 8·4 (0·56) | Ref | Ref | Ref | Ref |
|  | 3 | 51 | 18·4 (0·32) | 9·96 (0·58) |  | 54 | 17·8 (0·31) | 9·41 (0·57) | 0·40 (0·78) | 0·61 | 0·68 |
|  | 12 | 51 | 17·2 (0·49) | 8·81 (0·71) |  | 54 | 17·1 (0·47) | 8·70 (0·70) | 0·17 (0·78) | 0·83 | 0·83 |
| FIM locomotion  (2-14) | 0 | 51 | 5·19 (0·34) | Ref |  | 54 | 5·19 (0·34) | Ref | Ref | Ref | Ref |
|  | 3 | 51 | 11·99 (0·23) | 6·80 (0·37) |  | 54 | 11·51 (0·22) | 6·32 (0·37) | 0·43 (0·51) | 0·40 | 0·53 |
|  | 12 | 51 | 11·19 (0·37) | 6·00 (0·48) |  | 54 | 11·32 (0·36) | 6·13 (0·47) | -0·19 (0·51) | 0·71 | 0·83 |
| FIM communication  (2-14) | 0 | 51 | 13·5 (0·09) | Ref |  | 54 | 13·5 (0·09) | Ref | Ref | Ref | Ref |
|  | 3 | 51 | 13·9 (0·08) | 0·34 (0·12) |  | 54 | 13·7 (0·08) | 0·16 (0·12) | 0·15 (0·24) | 0·55 | 0·65 |
|  | 12 | 51 | 13·1 (0·26) | -0·49 (0·27) |  | 54 | 12·3 (0·25) | -1·24 (0·26) | 0·77 (0·24) | **0·0018** | 0·034 |
| FIM psychosocial  (3-21) | 0 | 51 | 19·9 (0·19) | Ref |  | 54 | 19·9 (0·19) | Ref | Ref | Ref | Ref |
|  | 3 | 51 | 20·3 (0·21) | 0·39 (0·27) |  | 54 | 20·2 (0·20) | 0·33 (0·26) | 0·03 (0·39) | 0·94 | 0·94 |
|  | 12 | 51 | 19·5 (0·35) | -0·36 (0·39) |  | 54 | 18·5 (0·34) | -1·40 (0·38) | 1·03 (0·39) | **0·0084** | 0·079 |
| Emotional status  (HADS, 0-42) | 0 | 51 | 18·9 (0·85) | Ref |  | 54 | 15·3 (0·57) | Ref | Ref | Ref | Ref |
|  | 3 | 51 | 15·3 (0·57) | -3·65 (0·76) |  | 54 | 14·1 (0·63) | -1·20 (0·75) | -2·36 (0·96) | **0·015** | 0·048 |
|  | 12 | 51 | 11·6 (0·65) | 0·27 (0·82) |  | 54 | 15·8 (0·55) | 0·46 (0·81) | -0·25 (0·96) | 0·79 | 0·83 |
| HADS anxiety  (0-21) | 0 | 51 | 7·61 (0·25) | Ref |  | 54 | 7·61 (0·25) | Ref | Ref | Ref | Ref |
|  | 3 | 51 | 7·60 (0·32) | -3·62 (0·56) |  | 54 | 7·54 (0·31) | -1·15 (0·55) | -2·40 (0·70) | **0·00071** | 0·0045 |
|  | 12 | 51 | 9·80 (0·29) | -1·90 (0·60) |  | 54 | 9·65 (0·28) | -1·56 (0·59) | -0·38 (0·70) | 0·59 | 0·83 |
| HADS depression  (0-21) | 0 | 51 | 7·66 (0·41) | Ref |  | 54 | 7·66 (0·41) | Ref | Ref | Ref | Ref |
|  | 3 | 51 | 4·03 (0·48) | -0·01 (0·36) |  | 54 | 6·51 (0·47) | -0·07 (0·35) | 0·08 (0·44) | 0·86 | 0·91 |
|  | 12 | 51 | 5·76 (0·42) | 2·19 (0·36) |  | 54 | 6·10 (0·41) | 2·05 (0·36) | 0·13 (0·44) | 0·77 | 0·83 |
| Pain  (NRS, 0-10) | 0 | 51 | 6·23 (0·20) | Ref |  | 54 | 6·23 (0·20) | Ref | Ref | Ref | Ref |
|  | 3 | 51 | 1·29 (0·28) | -4·94 (0·34) |  | 54 | 2·16 (0·27) | -4·07 (0·34) | -0·76 (0·40) | 0·060 | 0·13 |
|  | 12 | 51 | 2·01 (0·32) | -4·22 (0·37) |  | 54 | 2·14 (0·32) | -4·09 (0·36) | -0·10 (0·40) | 0·81 | 0·83 |
| Fear of falling  (SFES, 7–28) | 0 | 51 | 19·8 (0·60) | Ref |  | 54 | 19·8 (0·60) | Ref | Ref | Ref | Ref |
|  | 3 | 51 | 12·1 (0·78) | -7·73 (0·96) |  | 54 | 13·3 (0·76) | -6·51 (0·94) | -1·21 (1·10) | 0·27 | 0·43 |
|  | 12 | 51 | 12·7 (0·74) | -7·06 (0·94) |  | 54 | 13·8 (0·72) | -6·00 (0·92) | -1·02 (1·10) | 0·35 | 0·83 |
| Quality of life  (EQ5D, -0·65–1) | 0 | 51 | 0·299 (0·05) | Ref |  | 54 | 0·299 (0·05) | Ref | Ref | Ref | Ref |
|  | 3 | 51 | 0·408 (0·06) | 0·11 (0·07) |  | 54 | 0·317 (0·06) | 0·02 (0·07) | 0·09 (0·01) | 0·31 | 0·45 |
|  | 12 | 51 | 0·491 (0·06) | 0·19 (0·07) |  | 54 | 0·530 (0·06) | 0·23 (0·07) | -0·03 (0·01) | 0·71 | 0·83 |
| Self-perceived health  (EQ5D-VAS, 0-100) | 0 | 51 | 54·6 (2·21) | Ref |  | 54 | 54·6 (2·21) | Ref | Ref | Ref | Ref |
|  | 3 | 51 | 80·0 (2·12) | 25·42 (2·69) |  | 54 | 72·2 (2·06) | 17·53 (2·64) | 8·09 (3·72) | **0·030** | 0·081 |
|  | 12 | 51 | 71·5 (2·86) | 16·89 (3·44) |  | 54 | 65·0 (2·78) | 10·35 (3·37) | 7·77 (3·72) | **0·037** | 0·23 |
| EQ5D: EuroQol-5D; FDR: False Discovery Rate; FIM= Functional Independence Measure; HADS: Hospital Anxiety and Depression Scale; HG: Handgrip; *n*=sample size; NRS= Numeric rating scale; SE: Standard Error SFES-I= Short Falls Efficacy Scale; SPPB: Short Physical Performance Battery. Significant differences (p < 0·05) are highlighted in bold. | | | | | | | | | | | |

| **Table S5.** Differences in family caregivers' outcomes between groups at 3-month after surgery (post intervention) and at 1-year follow-up: sensitivity analyses | | | | | | | | | | | |
| --- | --- | --- | --- | --- | --- | --- | --- | --- | --- | --- | --- |
|  |  | ActiveHip+ telerehabilitation | | |  | Usual care rehabilitation | | |  |  |  |
| Outcome | Months | n | Mean (SE) | Change from  Baseline, Mean (SE) |  | n | Mean (SE) | Change from  Baseline, Mean (SE) | Differences in change from baseline  (ActiveHip+ vs· Usual care), Mean (SE) | *p*_uncorrected_ | *p*_FDR_ |
| Caregiver’ burden  (CSI, 0-13) | 0 | 51 | 6·36 (0·18) | Ref |  | 54 | 6·36 (0·18) | Ref | Ref | Ref | Ref |
|  | 3 | 51 | 3·64 (0·33) | -2·72 (0·35) |  | 54 | 4·59 (0·33) | -1·77 (0·34) | 0·96 (0·47) | **0·033** | 0·15 |
|  | 12 | 51 | 3·49 (0·40) | -2·87 (0·43) |  | 54 | 0·33 (0·38) | -2·68 (0·42) | 0·01 (0·45) | 0·97 | 0·97 |
| Emotional status  (HADS, 0-42) | 0 | 51 | 12·44 (0·42) | Ref |  | 54 | 12·44 (0·42) | Ref | Ref | Ref | Ref |
|  | 3 | 51 | 9·24 (0·54) | -3·20 (0·68) |  | 54 | 10·63 (0·52) | -1·81 (0·67) | 1·29 (0·73) | 0·076 | 0·22 |
|  | 12 | 51 | 16·03 (0·41) | 3·59 (0·57) |  | 54 | 15·44 (0·40) | 3·00 (0·56) | -0·60 (0·73) | 0·41 | 0·88 |
| HADS anxiety (0-21) | 0 | 51 | 3·94 (0·31) | Ref |  | 54 | 3·94 (0·31) | Ref | Ref | Ref | Ref |
|  | 3 | 51 | 2·07 (0·33) | -1·88 (0·46) |  | 54 | 2·44 (0·32) | -1·50 (0·45) | 0·29 (0·50) | 0·56 | 0·72 |
|  | 12 | 51 | 7·14 (0·29) | 3·20 (0·42) |  | 54 | 7·01 (0·28) | 3·07 (0·42) | -0·18 (0·50) | 0·72 | 0·96 |
| HADS depression (0-21) | 0 | 51 | 8·50 (0·20) | Ref |  | 54 | 8·50 (0·20) | Ref | Ref | Ref | Ref |
|  | 3 | 51 | 7·18 (0·34) | -1·31 (0·38) |  | 54 | 8·18 (0·33) | -0·32 (0·38) | 1·01 (0·39) | **0·010** | 0·090 |
|  | 12 | 51 | 8·82 (0·21) | 0·33 (0·28) |  | 54 | 8·37 (0·20) | -0·12 (0·27) | -0·42 (0·39) | 0·29 | 0·88 |
| Quality of life  (EQ5D, -0·65–1) | 0 | 51 | 0·83 (0·03) | Ref |  | 54 | 0·83 (0·03) | Ref | Ref | Ref | Ref |
|  | 3 | 51 | 0·90 (0·03) | 0·07 (0·04) |  | 54 | 0·86 (0·03) | 0·04 (0·04) | -0·03 (0·04) | 0·43 | 0·72 |
|  | 12 | 51 | 0·87 (0·03) | 0·04 (0·04) |  | 54 | 0·91 (0·03) | 0·08 (0·04) | 0·03 (0·04) | 0·49 | 0·88 |
| Health today  (EQ5D-VAS, 0-100) | 0 | 51 | 73·20 (2·24) | Ref |  | 54 | 73·20 (2·24) | Ref | Ref | Ref | Ref |
|  | 3 | 51 | 85·40 (1·46) | 12·20 (2·45) |  | 54 | 84·20 (1·42) | 11·05 (2·43) | -1·25 (3·00) | 0·68 | 0·77 |
|  | 12 | 51 | 84·70 (1·24) | 11·58 (2·54) |  | 54 | 83·00 (1·20) | 9·88 (2·52) | -2·25 (3·00) | 0·45 | 0·88 |
| Low back pain  (ODI, 0-50) | 0 | 51 | 9·90 (1·26) | Ref |  | 54 | 9·90 (1·26) | Ref | Ref | Ref | Ref |
|  | 3 | 51 | 5·45 (1·43) | -4·46 (1·87) |  | 54 | 6·63 (1·39) | -3·27 (1·83) | 1·45 (2·34) | 0·53 | 0·72 |
|  | 12 | 51 | 12·14 (1·87) | 2·24 (2·14) |  | 54 | 12·79 (1·82) | 2·88 (2·09) | 0·74 (2·34) | 0·75 | 0·96 |
| Fear of falling  (SFES-I, 7-28) | 0 | 51 | 20·80 (0·60) | Ref |  | 54 | 20·80 (0·60) | Ref | Ref | Ref | Ref |
|  | 3 | 51 | 11·20 (0·73) | -9·63 (0·91) |  | 54 | 11·90 (0·71) | -8·92 (0·89) | 0·76 (1·03) | 0·46 | 0·72 |
|  | 12 | 51 | 11·50 (0·66) | -9·38 (0·81) |  | 54 | 11·40 (0·64) | -9·44 (0·79) | -0·05 (1·03) | 0·96 | 0·97 |
| Self-reported fitness (IFIS,4–20) | 0 | 51 | 17·10 (0·33) | Ref |  | 54 | 17·10 (0·33) | Ref | Ref | Ref | Ref |
|  | 3 | 51 | 17·60 (0·40) | 0·47 (0·48) |  | 54 | 17·50 (0·39) | 0·39 (0·47) | -0·08 (0·57) | 0·89 | 0·89 |
|  | 12 | 51 | 17·40 (0·36) | 0·21 (0·49) |  | 54 | 16·60 (0·35) | -0·55 (0·48) | -0·73 (0·57) | 0·20 | 0·88 |
| CSI: Caregivers’ Strain Index; EQ5D: EuroQol-5D; FDR: False Discovery Rate; HADS: Hospital Anxiety and Depression Scale; IFIS: International Fitness Scale; *n*=sample size; ODI: Oswestry Low Back Disability; SE: Standard Error; SFES-I= Short Falls Efficacy Scale· Significant differences (p < 0·05) are highlighted in bold. | | | | | | | | | | | |
