# Supplementary material for: Effects of the ActiveHip+ mHealth intervention on the recovery of older adults with hip fracture and their family caregivers: a multicentre open-label randomised controlled trial
Source: eClinicalMedicine. 2024 Jun 7;73:102677. doi: 10.1016/j.eclinm.2024.102677 (PMC11192790; doi:10.1016/j.eclinm.2024.102677)
Supplement: TranslatedAbstract [file mmc2.docx]

*The following translations in Spanish were submitted by the authors and we reproduce them as supplied. They have not been peer-reviewed. Our editorial processes have only been applied to the original abstract in English, which should serve as reference for this manuscript.*”

# **Resumen**

**Introducción** Los sistemas de salud móvil (mHealth) son una alternativa prometedora para la rehabilitación de la fractura de cadera, que permite hacer frente a los limitados recursos sanitarios. La mitad de los adultos mayores no logra recuperar sus rutinas previas a la fractura, lo que impone una carga a los cuidadores. El objetivo de este estudio era probar la eficacia del sistema de salud móvil ActiveHip+ durante 3 meses sobre los resultados físicos y psicológicos de los adultos mayores con fractura de cadera y sus cuidadores familiares.

**Metodología** En un ensayo multicéntrico, abierto, aleatorizado y controlado realizado en 3 hospitales de Andalucía (España), se reclutó a pacientes mayores de 65 años con fractura de cadera, que previamente eran independientes y carecían de deterioro cognitivo, junto con sus cuidadores. Los participantes fueron asignados aleatoriamente (1:1) al grupo de intervención (ActiveHip+) o al grupo de control (atención estándar). El grupo de intervención se sometió a un programa de 12 semanas de educación sanitaria y tele-rehabilitación a través de la intervención ActiveHip+ mHealth. La variable principal, el rendimiento físico, se evaluó mediante la Short Physical Performance Battery en tres momentos temporales: al alta hospitalaria (basal), 3 meses después de la cirugía (post-intervención) y 1 año después de la cirugía. Los análisis primarios de los resultados primarios y los datos de seguridad siguieron un enfoque de intención de tratar. Este estudio está registrado en ClinicalTrials.gov, NCT04859309.

**Hallazgos** Entre el 1 de junio de 2021 y el 30 de junio de 2022 se analizaron los datos de 105 pacientes y sus cuidadores. Los pacientes que participaron en la intervención ActiveHip+ mHealth (media 7·11 puntos, SE 0·33) mostraron un mejor rendimiento físico en comparación con los pacientes del grupo de control (media de 5·71 puntos, SE 0·32) a los 3 meses de la cirugía (diferencia media en el cambio respecto al valor inicial 1·40 puntos, SE 0·36; puncorrected = 0·00011). Estos beneficios no se mantuvieron en el seguimiento de 1 año después de la cirugía (diferencia media en el cambio respecto al valor basal 0·19 puntos, SE 0·47; puncorrected = 0·68). No hubo eventos adversos como caídas y refracturas durante las sesiones de tele-rehabilitación. A los 3 meses, el grupo de intervención sufrió 2 caídas, frente a 4 en el grupo de control, sin que se observaran refracturas. Al año, el grupo de intervención experimentó 7 caídas y 1 refractura, mientras que hubo 13 caídas y 2 refracturas en el grupo de control.´

**Interpretación** Este estudio sugiere que la intervención ActiveHip+ mHealth puede ser eficaz para recuperar el rendimiento físico en adultos mayores con fractura de cadera. Es importante destacar que la implementación de ActiveHip+ en la práctica clínica diaria puede ser factible y ya se ha adoptado en 18 hospitales, la mayoría en España, pero también en Bélgica y Portugal. Así pues, ActiveHip+ podría ofrecer una solución prometedora cuando los recursos de rehabilitación son limitados. Sin embargo, su dependencia del apoyo del cuidador y la exclusión de participantes con deterioro cognitivo hacen que sea necesario ser cautelosos sobre su aplicabilidad. Además, el hecho de que no se mantuviera la eficacia en el seguimiento de 1 año pone de manifiesto la necesidad de perfeccionar la intervención ActiveHip+ para promover cambios conductuales duraderos.

**Keywords**, educación sanitaria, educación del paciente, fractura osteoporótica de cadera, salud digital, tele-rehabilitación.
